# Bypasses in intracellular glucose metabolism in iron‐limited *Pseudomonas putida*

**DOI:** 10.1002/mbo3.287

**Published:** 2015-09-16

**Authors:** Samantha S. Sasnow, Hua Wei, Ludmilla Aristilde

**Affiliations:** ^1^ Department of Biological and Environmental EngineeringCollege of Agricultural and Life SciencesCornell UniversityIthacaNew York14853

**Keywords:** *Pseudomonas*, glucose metabolism, iron limitation, gluconate, pyoverdine, siderophore

## Abstract

Decreased biomass growth in iron (Fe)‐limited *Pseudomonas* is generally attributed to downregulated expression of Fe‐requiring proteins accompanied by an increase in siderophore biosynthesis. Here, we applied a stable isotope‐assisted metabolomics approach to explore the underlying carbon metabolism in glucose‐grown *Pseudomonas putida* KT2440. Compared to Fe‐replete cells, Fe‐limited cells exhibited a sixfold reduction in growth rate but the glucose uptake rate was only halved, implying an imbalance between glucose uptake and biomass growth. This imbalance could not be explained by carbon loss via siderophore production, which accounted for only 10% of the carbon‐equivalent glucose uptake. In lieu of the classic glycolytic pathway, the Entner–Doudoroff (ED) pathway in *Pseudomonas* is the principal route for glucose catabolism following glucose oxidation to gluconate. Remarkably, gluconate secretion represented 44% of the glucose uptake in Fe‐limited cells but only 2% in Fe‐replete cells. Metabolic ^13^C flux analysis and intracellular metabolite levels under Fe limitation indicated a decrease in carbon fluxes through the ED pathway and through Fe‐containing metabolic enzymes. The secreted siderophore was found to promote dissolution of Fe‐bearing minerals to a greater extent than the high extracellular gluconate. In sum, bypasses in the Fe‐limited glucose metabolism were achieved to promote Fe availability via siderophore secretion and to reroute excess carbon influx via enhanced gluconate secretion.

## Introduction

Environmental aerobic bacteria have to cope with limited iron (Fe) availability during organic carbon utilization. In aerobic matrices, Fe is present predominantly within Fe hydroxides and oxides, which have very low solubility (less than 10^−10^ mol/L) and thus limited bioavailability (Boukhalfa and Crumbliss 2002; Andrews et al. 2003; Braun and Hantke 2011). However, high cellular Fe (*μ*mol/L level) is required due to the presence of Fe‐bearing components (e.g., hemes and Fe‐sulfur cofactors) in proteins that are essential to respiration and metabolism (Vasil and Ochsner 1999; Kim et al. 2010; Lim et al. 2012; Folsom et al., 2014). The expression of Fe‐containing metabolic enzymes were shown to be decreased in Fe‐limited *Pseudomonas* (Somerville et al. 1999; Heim et al., 2003; Palma et al., 2003; Vasil 2007; Bronstein et al, 2008; Kim et al. 2010; Lim et al. 2012), a genus of aerobic bacteria that are ubiquitous in natural soils and waters. *Pseudomonas* species include plant growth‐promoting bacteria that provide beneficial compounds to plant roots and microbial communities in the rhizosphere (Molina et al. 2000; Avis et al. 2008), as well as bacteria that are pathogenic to plants, insects, and humans (Peix et al. 2009). Due to their metabolic diversity, the carbon metabolism of *Pseudomonas* has been the subject of several investigations, which were conducted primarily under Fe‐replete conditions (Fuhrer et al. 2005; del Castillo et al. 2007; Blank et al. 2008; Puchalka et al. 2008; Lien et al. 2013; Sudarsan et al. 2014). However, studies are lacking on the consequence of Fe limitation on the metabolism of organic substrates by soil *Pseudomonas*.

**Figure 1:**
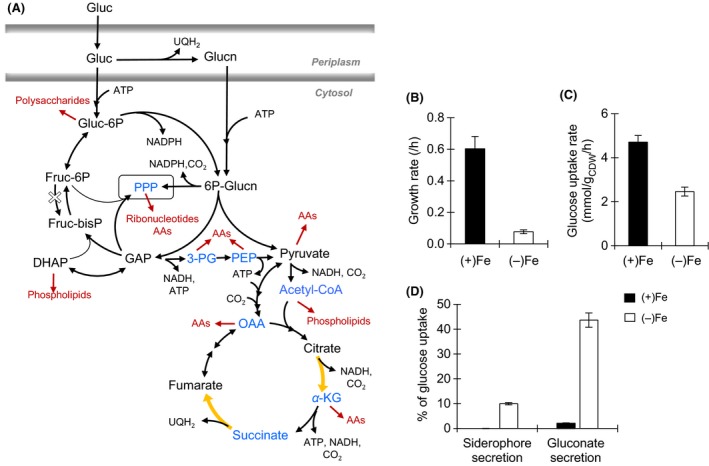
Schematic of metabolic pathways in *Pseudomonas putida*, growth phenotype, sugar consumption, and carbon secretion. (A) The metabolic routes through the Entner–Doudoroff pathway, the pentose phosphate pathway (PPP), and the tricarboxylic acid cycle are shown in black, the metabolites or pathways that are involved in siderophore biosynthesis are shown in blue, the anabolic pathways toward biomass production are shown in red, and location of Fe‐containing enzymes are shown in yellow. Gluconate, Glucn; 6‐phospho‐gluconate, 6P‐Glucn; glucose‐6‐phosphate, G6P; fructose‐6‐phosphate, F6P; fructose‐1,6‐bisphosphate, FBP; dihydroxyacetone‐phosphate, DHAP; glyceraldehyde‐3‐phosphate, GAP; 3‐PG; phosphoenolpyruvate, PEP; acetyl coenzyme A, acetyl‐CoA; adenosine triphosphate, ATP; reduced ubiquinone, UQH_2_; reduced nicotinamide adenosine, NADH; carbon dioxide, CO_2_; amino acids, AAs. (B) Growth rate of exponentially growing *P. putida* cells under Fe‐replete [Fe(+)] and Fe‐limiting [Fe(−)] conditions. (C) Glucose uptake rate. (D) Major sources of excreted carbons. See the text and Table S2 for more details. Values were calculated out of total carbon‐equivalent glucose uptake (from Fig. 2B): 28.26 ± 1.78 mmol C g_CDW_^−1^ h^−1^ and 14.77 ± 1.95 mmol C g_CDW_^−1^ h^−1^, respectively, in (+)Fe and (−)Fe growth media. The measured data (average ± standard deviation) were from four biological replicates. Two‐tailed unpaired *t‐*test analysis comparing the measured values for the two Fe conditions: *P* < 0.05.

Under Fe limitation, *Pseudomonas* are well known to secrete high‐affinity Fe‐binding molecules (or siderophores), which are metabolite expensive (Cézard et al. 2015). The biosynthesis of siderophores is derived from several pathways in the central carbon metabolism (Hider and Kong 2010) (Fig. 1A). In particular, pyoverdine (PVD), the primary siderophore produced by *Pseudomonas*, is a peptidic siderophore that contains various amino acids (Cézard et al. 2015). In addition, the Fe‐binding moieties in PVD are derived from different metabolites that feed into the pentose‐phosphate pathway (PPP) and the tricarboxylic acid (TCA) cycle. The catecholate moiety is synthesized from chorismate, a precursor to aromatic amino acids, that is synthesized from combining the metabolites erythrose‐4‐phosphate (from PPP) and phosphoenolpyruvate (PEP, from upstream of the TCA cycle); the hydroxamate moiety is synthesized from *α*‐ketoglutarate (*α*‐KG), a TCA cycle metabolite (Hider and Kong 2010) (Fig. 1A). Therefore, consumption of precursor metabolites during siderophore biosynthesis may contribute significantly to compromised biomass growth under Fe limitation.

However, decreased metabolic growth rate of Fe‐limited *Pseudomonas* has been attributed primarily to the decreased expression and activity of Fe‐containing respiratory proteins and several TCA cycle enzymes, including the Fe‐bearing enzymes aconitase and succinate dehydrogenase (Somerville et al. 1999; Vasil 2007; Kim et al. 2009, 2010; Lim et al. 2012). Previous studies with Fe‐limited *Escherichia coli* (Folsom et al. 2014) and *Saccharomyces cerevisiae* (Shakoury‐Elizeh et al. 2010) have also reported downregulation of TCA cycle enzymes (Folsom et al. 2014). In addition, enhanced secretion of metabolite by‐products in Fe‐limited *E. coli* was proposed to be the result of a shift in metabolism in order to avoid Fe‐demanding metabolic pathways (Folsom et al. 2014). This phenomenon of overflow metabolism has been implicated as a cellular strategy to deal with an imbalance between substrate uptake rates and those for energy production (Cornish‐Bowden 2013; Reaves et al. 2013). Under Fe‐sufficient conditions, glucose‐grown *Pseudomonas putida* has been shown to secrete low levels of gluconate and 2‐ketogluconate, the periplasmic oxidized products of glucose (del Castillo et al. 2007; Tlemçani et al. 2008). However, overflow secretion of metabolites has not been characterized in *Pseudomonas* within the context of dealing with shifted metabolism in response to Fe limitation.

In the present study, we seek to investigate the effects of Fe limitation on glucose metabolism in *P. putida* KT2440, a model environmental bacterium. Glucose was chosen here because this carbohydrate is an important environmentally relevant organic substrate. Glucose is a breakdown product of cellulosic materials in soils and is a common component of polysaccharides in plant secretions and root exudates. In *Pseudomonas*, glucose is metabolized through the Entner–Doudoroff (ED) pathway by splitting 6‐phosphogluconate (6P‐Glucn), instead of the classic Embden–Meyerhof–Parnas (EMP) glycolytic pathway that splits fructose‐1,6‐bisphosphate (FBP) (Fig. 1A).

Once taken up by the cells, glucose is either phosphorylated directly to glucose‐6‐phosphate (G6P) in the cytosol or oxidized to gluconate (and to a lesser extent 2‐ketogluconate) in the periplasm (Tlemçani et al. 2008) (Fig. 1A). The EMP is not fully functional in *Pseudomonas* because the enzyme that catalyzes the eventual conversion of G6P to FBP is lacking (Poblete‐Castro et al. 2012) (Fig. 1A). Glucose thus enters the central carbon metabolism through 6P‐Glucn, following the phosphorylation of periplasmic gluconate to 6P‐Glucn or the oxidation of cytosolic G6P to 6P‐Glucn (Fig. 1A). In the ED pathway, 6P‐Glucn is converted to pyruvate and glyceraldehyde‐3‐phosphate (GAP) (Fig. 1A). Both 6P‐Glucn and the ED pathway‐generated metabolites are precursors to pentose phosphates, which are required for ribonucleotide and amino acid precursors in the PPP (Fig. 1A). Subsequent metabolism downstream of the ED pathway and through the TCA cycle produces more amino acid precursors and energy‐rich molecules (Fig. 1A). Therefore, influx of glucose carbons toward 6P‐Glucn and the ED pathway is a prerequisite to the biosynthesis of biomass precursors in the central carbon metabolism during growth on glucose.

Based on the aforementioned studies, we hypothesized that the decreased metabolic investment toward biomass growth in glucose‐grown *P. putida* under Fe limitation could be due to (1) a decrease in carbon uptake and subsequent influx toward the ED pathway, (2) a decrease in metabolic flux through Fe‐demanding pathways, (3) overflow secretion of partially oxidized metabolites, and (4) a high metabolic expense for siderophore biosynthesis. Using liquid chromatography–mass spectrometry (LC‐MS), we applied a stable isotope‐assisted metabolomics approach to explore these hypotheses. We found that glucose uptake did not agree with the significant reduction in the growth rate of Fe‐limited cells. And, the siderophore secretion rate did not represent a significant carbon loss. Our metabolic analysis revealed that glucose uptake was routed away from entry into the ED pathway through a significant increase in gluconate secretion. Monitoring of in vivo metabolic enzyme activity through kinetic flux profiling (KFP) demonstrated a decrease in the metabolic flux through the ED pathway as well as through Fe‐containing enzymes; this was confirmed via quantitative metabolic flux analysis (MFA).

## Materials and Methods

### Cultures, chemicals, and equipment

*Pseudomonas putida* cells (strain KT2440) were obtained from the American Type Culture Collection (Manassas, VA). Stable isotope‐labeled glucose was obtained from Cambridge Isotopes. All other chemicals were obtained analytical grade from Fisher Scientific (Pittsburg, PA, USA) or Sigma‐Aldrich (St Louis, MO, USA). All solutions were prepared with ultrapure water (18.2 MΩ·cm, Millipore; Billerica, MA, USA) or LC‐MS water. All containers used for media storage, culturing, and mineral dissolution were acid washed (15% and HNO_3_) overnight, washed three times with ultrapure water, and autoclaved. Nylon filters (0.2 *μ*m) were used for all filtration. Absorbance measurements were conducted using an Agilent Cary UV–visible spectrophotometer. The LC‐MS analysis was performed with ultra‐high performance LC [Thermo Scientific (Waltham, MA, USA) DionexUltimate 3000] coupled with a high‐resolution MS (Thermo Scientific *Q Exactive* quadrupole‐Orbitrap hybrid MS). Total dissolved Fe in solution was determined via inductively coupled plasma atomic emission spectroscopy (ICP‐AES, Spectro Analytical, detection limit = 36 nmol/L).

### Culturing

To facilitate high reproducibility of the experiments, we focused our study on exponentially growing batch cultures. As previously demonstrated with *Pseudomonas syringae* (Kim et al. 2010), achieving reproducible chemostat conditions of Fe‐limited *Pseudomonas* cultures has proven to be challenging due to the dynamic response of the Fe‐limited cells as a function of initial cell density and of dilution rate. Here, batch liquid culturing of *P. putida* KT2440 was conducted in an incubator (model I24; New Brunswick Scientific, Edison, NJ) kept at 30°C. Liquid cultures, in either 250‐mL Erlenmeyer flasks or 20‐mL glass test tubes, were agitated vigorously on a rotary shaker (200 rpm) in order to obtain well‐mixed aerated cultures. Cells were first grown in Luria–Bertani medium and subsequently transferred to minimal growth medium containing glucose as the sole organic carbon source. The growth medium, which was pH‐adjusted (7.0) and filter‐sterilized, contained the following: 20 mmol/L K_2_HPO_4_, 5 mmol/L NaH_2_PO_4_, 0.8 mmol/L MgSO_4_·7H_2_O, 37 mmol/L NH_4_Cl, 34 *μ*mol/L CaCl_2_·2H_2_O, 13 *μ*mol/L CuSO_4_·5H_2_O, 0.49 *μ*mol/L H_3_BO_3_, 35 *μ*mol/L ZnSO_4_·5H_2_O, 2.9 *μ*mol/L MnSO_4_·5H_2_O, 0.11 *μ*mol/L NiCl_2_·5H_2_O, 0.6 *μ*mol/L Na_2_MoO_4_·5H_2_O, and 51.3 mmol/L (or 9.25 g/L) of glucose. For the Fe‐replete condition, the growth medium was supplemented with 20 *μ*mol/L FeSO_4_·7H_2_O. For the Fe‐limiting condition, the growth medium was not supplemented with Fe of any forms; the background Fe concentration of the minimal medium (including all the salts and the organic substrate) was below the Fe detection limit as measured by ICP‐AES. Subjecting the cells to at least two transfers into fresh minimal growth medium (with or without Fe supplementation) ensured that the cells were acclimated to the respective growth medium. As was done previously (Lim et al. 2012), we opted not to use exogenous chelators in our growth media because natural molecules with multiple carboxylic acids such as citrate can be utilized as an organic substrate by *Pseudomonas* (Mandalakis et al. 2013) and synthetic chelators such as ethylenediamine tetra‐acetic acid (EDTA) have been shown to compromise growth of *Pseudomonas* (Banin et al. 2006). Over the sampled time course, the continuous detection of the siderophore PVD produced by the Fe‐limited cells indicated that the Fe‐limitation condition was maintained in the growth medium; there was no detected production of PVD by the cells grown in the Fe‐replete condition.

### Monitoring growth

Growth of the bacterial cells (four biological replicates) was monitored by measuring the optical density at 600 nm (OD_600_) (Fig. S1). Measurements of cell dry weight in grams (g_CDW_) as a function of OD_600_ were obtained following lyophilization of sample aliquots (1.5 mL) using a Labconco (Kansas City, MO, USA) Freeze‐Dryer System. A conversion factor of 0.58 ± 0.08 g_CDW_ L^−1^ per OD_600_ was determined from sampling during the exponential growth phase. This conversion factor is consistent with a previous value, 0.55 g_CDW_ L^−1^ per OD_660_, obtained with batch cultures of glucose‐grown *P. putida* mt‐2 (Tlemçani et al. 2008).

### Measurement of glucose uptake

Samples (four biological replicates) were harvested during the exponential growth of *P. putida* by filtering an aliquot (2 mL) and storing the filtrate at 4°C until NMR analysis. NMR samples were prepared as previously described (Aristilde et al. 2015). NMR spectra were recorded on a Varian Unity (Santa Clara, CA, USA) INOVA 600‐MHz NMR spectrometer. The depletion of glucose in the extracellular medium was taken as a surrogate to sugar uptake as done previously (Aristilde et al. 2015). We validated this assumption by conducting kinetic isotopic labeling, as described below. The glucose uptake rate in the exponentially growing cells was calculated by regression analysis.

### Metabolite quantitation

Samples (four biological replicates) were analyzed by reversed‐phase ion‐pairing LC coupled with electrospray ionization high‐resolution MS. The MS was operated in full scan negative mode (*m/z* range 70–900) for the detection of metabolites based on accurate masses, following established methods (Lu et al. 2010). For extracellular metabolites, samples were harvested at several time points throughout the exponential growth phase (Fig. S2), diluted 1:10 or 1:100, before analysis. Sodium benzoate was used as an internal standard. Excretion rates of metabolites were computed by regression analysis. The intracellular metabolites were determined as done previously (Lu et al. 2010). Briefly, an aliquot (3 mL) of the cultures was filtered and the metabolism of the cells was immediately quenched by placing the filters in a 2‐mL solution (4°C) of methanol:acetonitrile:water (40:40:20). The supernatants with the extracted metabolites were analyzed after the removal of lysed cell particulates via centrifugation. Metabolite identification was validated by using metabolite standards. Quantitation limits of metabolite levels, which were above their limit of detection, were set to the signal of the blank measurements for intracellular metabolites and the water blank for the extracellular metabolites. Metabolites, including the multiple isotopologue peaks, were identified and quantified using the Metabolomics Analysis and Visualization Engine (MAVEN) software package (Clasquin et al. 2012).

### Monitoring intracellular metabolism

Intracellular metabolism was monitored in exponentially growing cells wherein pseudosteady‐state conditions of intracellular metabolite pools (i.e., metabolite influx equates metabolite efflux in metabolic reactions) can be assumed (Yuan et al. 2008). To confirm the canonical metabolic pathways of glucose metabolism, long‐term isotopic enrichment of intracellular metabolites was conducted with [1,2‐^13^C_2_]‐glucose in the minimal growth medium. This was achieved by growing the *P. putida* cells (four biological replicates) for about two doubling times during early to mid‐exponential growth phase (at least 3 h for Fe‐replete cells and 12 h for Fe‐limiting cells) (Fig. S1). In previous metabolic labeling studies on *Pseudomonas*, the labeling of these metabolites were deduced from the labeling patterns of derivatized amino acids detected via gas chromatography–mass spectrometry (Fuhrer et al. 2005; del Castillo et al. 2007; Blank et al. 2008; Sudarsan et al. 2014). Using LC‐MS, we were able to determine the labeling patterns of metabolites directly involved in the core metabolic pathways. Therefore, our approach allowed for explicit elucidation of intracellular glucose metabolism upstream of amino acid biosynthetic pathways.

We used kinetic isotopic incorporation of [U‐^13^C_6_]‐glucose into intracellular metabolites in order capture *in vivo* fluxes through metabolic enzymes (Yuan et al. 2008). This thus served as a proxy for *in vivo* activity of these enzymes. The KFP was determined using established protocols (Yuan et al. 2008). Briefly, aliquots (3 mL) from liquid cultures growing on unlabeled glucose were filtered and transferred to media plates with unlabeled glucose. Following multiple doubling times, filter cultures were transferred to media plates containing [U‐^13^C] glucose. At specific time points after the transfer (30 sec, 1, 2, 5, 15, 30 min), metabolism was quenched using the aforementioned 40:40:20 solvent mixture and the extracts were prepared and analyzed as mentioned above.

The multiple isotopologues (different labeled forms of the same compound with the same number of ^13^C‐labeled carbons) resulting from the ^13^C labeling experiments were obtained for the following metabolites: 6P‐Glucn, G6P, fructose‐6‐phosphate (F6P), FBP, ribose‐5‐phosphate (R5P), xylulose‐5‐phosphate (Xu5P), sedoheptulose‐7‐phosphate (S7P), dihydroxyacetone‐phosphate (DHAP), 3‐phosphoglycerate (3‐PG), PEP, pyruvate, citrate, *α*‐KG, succinate, and fumarate. As done previously (Amador‐Noguez et al. 2010), the labeling of OAA was taken to be the same as the labeling of aspartate, the amino acid that is synthesized from OAA, by assuming that these two compounds were in equilibrium (Kishore et al. 1998). The multiple isotopologues were identified using the MAVEN software package (Clasquin et al. 2012). The ^13^C‐labeled fractions were corrected for the natural ^13^C abundance.

To determine the intracellular levels of selected metabolites, a previously described isotope ratio‐based procedure was employed (Bennett et al. 2008). Briefly, cells were grown as described above with [U‐^13^C_6_]‐glucose in the minimal medium to achieve near‐complete isotopic enrichment of the intracellular metabolome. Metabolites were extracted as described above and samples were spiked with known concentrations of unlabeled standards prior to analysis. After correcting for extracellular level, the intracellular level of metabolites was calculated using the ratio of fully labeled metabolites to the nonlabeled internal standard, following corrections for natural ^13^C abundance. Unspiked samples were also examined for nonlabeled fractions to determine whether further corrections were necessary (Bennett et al. 2008). Metabolite levels were normalized to cell mass at the time of sampling.

### Siderophore quantitation

The amount of the siderophore PVD was determined in pH‐adjusted (pH 7) bacterial supernatants (three biological replicates) via absorbance (400 nm), as described previously (Ferret et al. 2014), and using a PVD standard (Sigma‐Aldrich) (quantitation limit = 0.84 *μ*mol/L). In addition to exhibiting similar absorbance profiles, we have verified via LC‐MS that both the standard and the secreted PVD possessed the same dihydroxyquinoline‐type chromophore (Wei and Aristilde 2015). We also determined that there was no change in the absorbance signal at 400 nm in the presence of either Fe^2+^ or Fe^3+^ in solution (at both 50 nmol/L and 50 *μ*mol/L total Fe), within an error precision of two standard deviations of the PVD quantitation (Fig. S10). The PVD excretion rate of the exponentially growing liquid cultures was calculated by regression analysis.

### Metabolic flux modeling

Metabolic flux balance analysis was constrained using the following experimental data (averaged values ± standard deviation): glucose uptake rate, metabolite and siderophore excretion rates, labeling patterns of metabolites from the long‐term labeling experiment with [1,2‐^13^C_2_]‐glucose, and calculated effluxes of metabolites toward biomass production. Sugar uptake rates and metabolite excretion rates were measured using NMR analysis and LC‐MS analysis of the extracellular media, respectively, as described above. The growth rate under each condition was used to calculate effluxes toward biomass production from glycolytic, PPP, TCA cycle metabolites (Winsor et al. 2011) based on the biomass composition of *P. putida* (protein, RNA, DNA, lipids, and carbohydrates) (Van Duuren et al. 2013) and *E. coli* (peptidoglycan) (Glauner et al. 1988; Feist et al. 2007) (Table S6). We applied the software suite 13CFLUX2 package (http://www.13cflux.net) (Weitzel et al. 2012) to set up the modeled metabolic network of reactions as well as obtaining initial metabolic fluxes for further optimization. The model‐estimated labeling patterns from the modeling of the stoichiometric reaction network were optimized on the measured labeling patterns.

### Mineral dissolution

Dissolution experiments (10 mL) were conducted in 50 mL propylene tubes with the following minerals at 1 g L^−1^: hematite (Fe_2_O_3_), goethite [FeO(OH)], or magnetite (Fe_3_O_4_). These experiments (two to six independent replicates) were conducted with cell‐free extracts obtained when the growth of cells reached the end of exponential phase under both Fe conditions. Cells were removed by centrifugation (21,130*g*, 5 min) followed by filtering. Control experiments were conducted with the minimal growth medium (without Fe supplementation) alone or amended with glucose (100 mmol/L), gluconate (10 mmol/L), or PVD standard (100 *μ*mol/L). Prior to the mineral addition, all solutions were adjusted to pH 7.5. Experiments were conducted in an incubator shaker as described above for the cell culturing. The tubes were wrapped in aluminum foil to exclude light. Samples were taken after 100 h reaction time, centrifuged, and filtered. Total Fe was measured via ICP‐AES analysis. Prior to analysis, sample pH was adjusted to pH 4.5 with acetate buffer. Metal concentrations were blank‐corrected with appropriate blank experiments: minerals without cell extracts or minimal medium without minerals.

## Results and Discussion

### Growth phenotype and sugar consumption

To investigate the link between biomass growth and carbon uptake under Fe limitation, we obtained the exponential growth rate and the glucose uptake rate in *P. putida* under both Fe‐replete and Fe‐limited conditions (Figs. 1B, C, and S1). The growth rate obtained with the Fe‐replete *P. putida* cells was 0.60 ± 0.04 h^−1^, in close agreement with previous values obtained with Fe‐replete glucose‐grown *Pseudomonas fluorescens* (Fuhrer et al. 2005) and *P. putida*, (del Castillo et al., 2007) (0.49 ± 0.03 h^−1^ and 0.56 ± 0.02 h^−1^, respectively) (Fig. 1B). The growth rate of the Fe‐limited cells, 0.08 ± 0.02 h^−1^, was seven times slower than the Fe‐replete value (Fig. 1B). Upon the addition of Fe (30 *μ*mol/L) to the extracellular medium, growth of the Fe‐limited cells was quickly recovered to similar growth kinetics as the Fe‐replete cells (Fig. S2). This confirmed that the reduced growth rate of the cells was due to Fe limitation.

The glucose uptake rate did not correlate positively with the reduced biomass growth (Fig. 1C). The Fe‐replete glucose uptake rate was 4.71 ± 0.30 mmol g_CDW_^−1^ h^−1^, consistent with previously reported uptake rates in Fe‐replete *P. fluorescens* (Fuhrer et al. 2005) and *P. putida* (del Castillo et al., 2007) (4.79 ± 0.39 mmol g_CDW_^−1^ h^−1^ and 4.81 ± 0.17 mmol g_CDW_^−1^ h^−1^, respectively) (Fig. 1C). The Fe‐limiting glucose uptake rate, 2.46 ± 0.20 mmol g_CDW_^−1^ h^−1^, was only half of the Fe‐replete value, despite the sevenfold reduction in the growth rate of the Fe‐limited cells (Fig. 1C).

Our findings thus implied that the carbon influx in the Fe‐limited cells was in excess of the carbon investment toward biomass growth. The carbon‐equivalent uptake rate was calculated to be 28.26 ± 1.78 mmol C g_CDW_^−1^ h^−1^ and 14.77 ± 1.95 mmol C g_CDW_^−1^ h^−1^ for the Fe‐replete and Fe‐limited cells, respectively (Fig. 1C). Thus, the excess carbon influx in the Fe‐limited cells was 10.06 ± 1.65 mmol C g_CDW_^−1 ^h^−1^, representing 68.11 ± 1.94% of the glucose uptake rate under Fe limitation.

### Siderophore and metabolite secretion

As a first step in accounting for the imbalance between carbon uptake and biomass growth, we obtained the siderophore secretion rate. In contrast to other *Pseudomonas* strains, the *P. putida* strain (KT2440) used in this study does not secrete other specialized siderophores in addition to PVD (Cornelis 2010) – specialized siderophores exclude metabolite compounds (e.g., citrate) that are known to be strong Fe chelators. We measured a PVD secretion rate of 23.7 ± 4.4 *μ*mol g_CDW_^−1^ h^−1^ with the Fe‐limited cells; no PVD production was detected with the Fe‐replete cells (Table S1). We also determined that a total of 62 moles of carbons was consumed from the central carbon metabolism to produce each mole of PVD, based on the PVD structure produced by *P. putida* KT2440 (succinate–chromophore–aspartate–ornithine–hydroxyaspartate–diaminobutyrate–glycine–serine–cyclic ornithine) and the additional fatty acid chain needed to form the pre‐PVD molecule (Schalk and Guillon 2013; Wei and Aristilde 2015) (Table S1). Therefore, 1.47 ± 0.27 mmol C g_CDW_^−1^ h^−1^ was dedicated to the production of the secreted PVD, representing only 9.95 ± 0.47% of the total carbon influx in the Fe‐limited cells (Fig. 1D). Therefore, carbon loss via siderophore secretion was insufficient to account for the carbon imbalance in the Fe‐limited cells.

In order to account for the remaining carbon excess in Fe‐limited *P. putida*, we examined possible overflow secretions of metabolite by‐products. Under both Fe conditions, we obtained the secretion rates of several organic acids from the central carbon metabolism: gluconate, 6P‐Glucn, 3‐PG, PEP, pyruvate, citrate, *α*‐KG, glutamate, succinate, fumarate, malate, and aspartate (Table S2). Excluding gluconate, the sum of the metabolite secretion rates was 22.59 ± 6.63 *μ*mol C g_CDW_^−1^ h^−1^ and 30.28 ± 13.27 *μ*mol C g_CDW_^−1^ h^−1^ in the Fe‐replete and Fe‐limited cells, respectively; these rates represent less than 0.3% of the glucose uptake rate (Table S2). However, the gluconate secretion rate was at one to two orders of magnitude above the rates of the other metabolites, respectively, 0.57 ± 0.08 mmol C g_CDW_^−1^ h^−1^ and 6.44 ± 1.33 mmol C g_CDW_^−1^ h^−1^ in the Fe‐replete and Fe‐limited cells, respectively (Table S2). Thus, 43.6 ± 0.03% of the glucose taken up by Fe‐limited cells was secreted as gluconate, whereas the corresponding value in the Fe‐replete cells was only 2.01 ± 0.16% (Fig. 1D).

High levels of extracellular gluconate can be attributed in part to its formation in the periplasm from where it can be easily excreted (del Castillo et al. 2007; Tlemçani et al. 2008). Notably, the gluconate secretion rate in the Fe‐limited cells was 10 times higher than in the Fe‐replete cells, implying a specific response to Fe limitation. In accordance with the overflow metabolism described in the Introduction section, gluconate secretion may serve to relieve excess build up of metabolites in Fe‐limited metabolism in *P. putida*. A similar strategy was proposed to occur in Fe‐limited *E. coli* through the secretion of organic acids from lower glycolysis (Folsom et al., 2014). As illustrated in Fig. 1A, the periplasmic oxidation of glucose to gluconate by membrane‐bound dehydrogenases is coupled with the production of reduced ubiquinone. Thus, the production of gluconate in Fe‐limited *P. putida* contributes to the generation of reducing equivalents, while the enhanced glucose secretion would avoid the metabolic expense of its intracellular metabolism. [Correction added on 30 September 2015 after first online publication: This sentence has been newly added in this version].

Thus far, we were able to account for 53.58 ± 0.03% of the glucose uptake in Fe‐limited cells as carbon losses via, to a major extent, gluconate secretion and, to a lesser extent, siderophore secretion. However, additional losses are expected via carbon dioxide (CO_2_) efflux during glucose metabolism through the central carbon metabolism (Fig. 1A). Therefore, a more complete picture of the carbon balance would require elucidation of the intracellular metabolic fluxes.

### Elucidating the metabolic network structure

**Figure 2:**
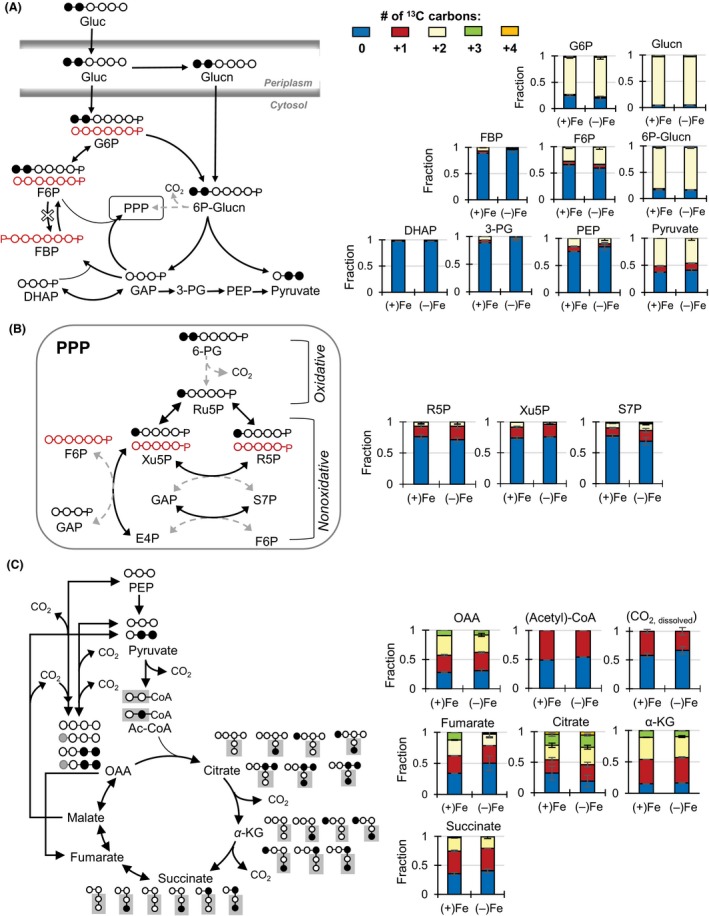
Elucidating the metabolic network structure through the assimilation of [1,2‐^13^C_2_]‐glucose into intracellular metabolites. (A) Carbon mapping (left) and metabolite labeling (right) in the periplasm, the Embden–Meyerhof–Parnas (EMP), and the Entner–Doudoroff (ED) pathways. The red‐colored arrows and carbon skeletons are to illustrate the formation of nonlabeled metabolites that are directly a result of the ED pathway into the reverse route of the EMP pathway. (B) Carbon mapping (left) and metabolite labeling (right) in the pentose‐phosphate pathway (PPP). (C) Carbon mapping (left) and metabolite labeling (right) in the tricarboxylic acid cycle (C). The broken‐lined arrows indicate the minor formation routes of the metabolites. Labeling patterns: nonlabeled (blue), singly labeled (red), doubly labeled (yellow), and triply labeled (green). Legend for metabolite names are the same as reported in Figure 1. Isotopologue data (average ± standard deviation) were obtained from four biological replicates. [Correction added on 30 September 2015 after first online publication: Figure 2 had inconsistent labelling and these have now been corrected in this version].

A metabolic network model was used to map the carbon fluxes through the periplasm, the ED pathway, the reverse EMP pathway, the PPP, and the TCA cycle. We confirmed the metabolic network structure through long‐term isotopic enrichment of intracellular metabolites by growing cells on [1,2‐^13^C_2_]‐glucose under both Fe conditions (Fig. 2).

Examination of the labeling patterns revealed that the ED pathway, rather than the forward EMP pathway, is the primary catabolic pathway of glucose in Fe‐limited *P. putida* following glucose entry into the cells. This agrees with similar conclusions obtained previously with Fe‐replete *P. putida* (Fuhrer et al. 2005; del Castillo et al. 2007; Blank et al. 2008; Sudarsan et al. 2014). High enrichment of doubly ^13^C‐labeled forms of both gluconate and G6P (on average, 90% and 75%, respectively) was consistent with the assimilation of the doubly ^13^C‐labeled glucose (Fig. 2A). On the other hand, up to 20% of G6P was in nonlabeled forms, indicating that there was another pathway forming this metabolite (Fig. 2A). In the ED pathway, one mole of [1,2‐^13^C_2_]‐6P‐Glucn would generate one mole of doubly ^13^C‐labeled pyruvate and one mole of nonlabeled GAP (Fig. 2A). Consistent with the ED pathway, we obtained a high fraction of nonlabeled DHAP (97% and more), along with the presence of doubly ^13^C‐labeled pyruvate, in both Fe‐replete and Fe‐limited *P. putida* cells (Fig. 2A). The labeling pattern of pyruvate (46–51% in doubly ^13^C‐labeled forms and 37–41% in nonlabeled forms) reflected the formation of pyruvate both from the splitting of doubly ^13^C‐labeled 6P‐Glucn through the ED pathway and from nonlabeled metabolites downstream of the nonlabeled GAP (Fig. 2A). The absence of the forward EMP pathway was further confirmed by the high fraction (over 95%) of nonlabeled FBP, compared to 60–65% of nonlabeled F6P (Fig. 2A), in accordance with the absence of the phosphofructokinase enzyme. On the other hand, the reverse EMP pathway produced the high fraction of nonlabeled forms of FBP via the combination of nonlabeled DHAP and nonlabeled GAP generated by the ED pathway (Fig. 2A).

With respect to the PPP, the labeling patterns of PPP metabolites (R5P, Xu5P, and S7P) indicated that these metabolites were formed primarily from the nonoxidative PPP, rather than from the oxidative PPP (Fig. 2B). Nonlabeled PPP metabolites would be produced through the nonoxidative PPP by making use of metabolites downstream of the ED pathway and the reverse EMP pathway (Fig. 2B). On the other hand, the decarboxylation of the doubly ^13^C‐labeled 6P‐Glucn through the oxidative PPP would produce primarily singly ^13^C‐labeled pentose phosphates (R5P and Xu5P). Nearly 80% of the PPP metabolites (R5P, Xu5P, and S7P) were in nonlabeled forms under both Fe conditions, indicating that the nonoxidative PPP was the primary PPP route and that the flux through the oxidative PPP was small (Fig. 2B). A small flux through the oxidative PPP was also reported previously for *P. putida* grown on glucose and other carbon sources under Fe‐replete conditions (Fuhrer et al. 2005; Blank et al. 2008; Sudarsan et al. 2014). The low contribution of the oxidative PPP implied that flux through the ED pathway played a controlling factor in carbon flux toward the rest of the central carbon metabolism, in addition to ribonucleotide biosynthesis (Fig. 1). This also implied that there would be very little CO_2_ efflux via the oxidative PPP.

The labeling patterns of metabolites downstream of the ED pathway and the TCA cycle were consistent with the canonical route of carbon flow through these pathways (Fig. 2C). Following decarboxylation, the pyruvate labeling produced nonlabeled and singly ^13^C‐labeled acetyl‐CoA (Figs. 2C, S3). The ^13^C labeling of citrate (nonlabeled, singly, doubly, triply, and minor quadruply labeled) was in agreement with the combination of acetyl‐CoA (nonlabeled and singly ^13^C labeled) and oxaloacetate (OAA) (nonlabeled, singly, doubly, and minor triply labeled) in the TCA cycle (Fig. 2C). The decarboxylation of citrate then resulted in primarily unlabeled, singly labeled, doubly labeled, and triply labeled *α*‐KG (Fig. 2C). Subsequent decarboxylation of *α*‐KG resulted in nonlabeled, singly labeled, and doubly labeled forms of succinate (Fig. 2C). Due to the two decarboxylation reactions between citrate and succinate, most labeled forms of citrate became singly ^13^C‐labeled succinate, which would eventually generate singly ^13^C‐labeled fumarate, malate, and OAA (Fig. 2C).

Thus, decarboxylation reactions during carbon metabolism would contribute labeled CO_2_, which was determined to be about 30% and 45% of the total dissolved CO_2_ pool in the Fe‐replete and Fe‐limited cells, respectively (Figs. 2C, S4). Therefore, singly ^13^C‐labeled OAA can result both from carboxylation reactions of metabolites (PEP and pyruvate) downstream of the ED pathway and from the TCA cycle downstream of succinate (Fig. 2C). The net contribution of these two fluxes would be resolved by conducting quantitative MFA, which will be discussed in the next section.

The ^13^C labeling pattern of fumarate, a TCA cycle metabolite, indicated a change in its biosynthetic flux in Fe‐limited cells. (Fig. 2C). Specifically, triply labeled fumarate was six times higher in Fe‐replete cells than in Fe‐limited cells (on average, 12% and 2%, respectively) (Fig. 2C). The biosynthesis of fumarate is from succinate during TCA cycle metabolism and from aspartate (an OAA‐derived amino acid) during ribonucleotide biosynthesis (Fig. 2C). The conversion of succinate to fumarate would maintain the same labeling pattern (Fig. 2C). The lack of triply labeled succinate indicated that this labeled form in fumarate was not from succinate (Fig. 2C). Although OAA had equal abundance of triply ^13^C‐labeled forms under both Fe conditions, we measured an enrichment of triply labeled fumarate in the Fe‐replete cells compared to the Fe‐limited cells, consistent with decreased *de novo* ribonucleotide biosynthesis due to the slower growth rate of Fe‐limited cells (Figs. 1B and  2C). Therefore, fumarate is produced primarily from the metabolic flux through the TCA cycle under Fe limitation, whereas this flux is supplemented by the biosynthetic flux of ribonucleotides under favorable Fe condition.

The glyoxylate shunt, which bypasses Fe‐containing enzymes in the TCA cycle, provides an alternate path to produce malate and succinate (Fig. S5). Minimal to no involvement of this pathway was determined during flux analysis of Fe‐replete glucose‐grown *P. putida* (Blank et al., 2008; Sudarsan et al. 2014). The findings from our labeling experiments also suggested that involvement of the glyoxylate shunt was insignificant under both Fe conditions (SI, Fig. S5).

In sum, examination of the labeling patterns of intracellular metabolites allowed us to map out the metabolic network structure. However, it would be challenging to determine the relative contributions of the different metabolic reactions based only on the metabolite labeling schemes. Therefore, we applied a MFA approach to quantitate the fluxes through the different metabolic pathways.

### Quantitative metabolic flux modeling

The MFA was constrained on the glucose uptake rate, siderophore secretion, metabolite secretion, and biomass growth. The agreement between model‐estimated and experimentally determined labeling patterns demonstrated the good quality of our optimization fits under both Fe conditions (Figs. S6 and S7). Using the metabolic network structure elucidated in the previous section, we were able to quantitate a total of 28 metabolic reaction rates in the central carbon metabolism (Table S3).

**Figure 3:**
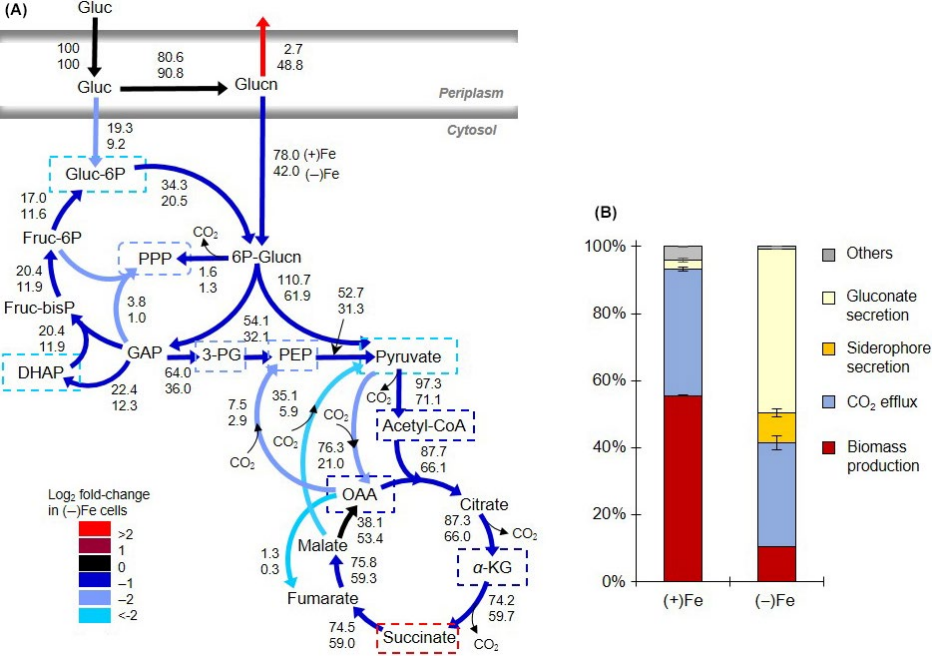
Quantitative metabolic flux analysis. (A) Metabolic flux modeling constrained by ^13^C metabolite labeling, metabolite secretion, siderophore secretion, and biomass composition. The top and bottom numbers represent, respectively, the metabolic reaction rates normalized to glucose uptake in Fe‐replete [(+)Fe] and the Fe‐limited [(−)Fe] *Pseudomonas putida* cells. Metabolites outlined with broken‐lined squares contribute to biomass and siderophore biosynthesis. The different colors reflect the log_2_ fold‐change of the reaction rates in the (−)Fe cells compared to those in (+)Fe cells. Statistics on the estimated reaction rates are presented in Table S3 and S4. (B) Percentage investment of the total glucose consumption rate toward gluconate secretion (yellow), siderophore secretion as pyoverdine (orange), net carbon dioxide efflux generated from metabolic reactions (blue), and biomass production (biosynthesis of amino acids, ribonucleotides, and cell membrane components) (dark red). Others denote the sum of other metabolite excretions. The values were estimated from the experimentally constrained metabolic flux analysis (illustrated in part A) on *P. putida* cells exponentially growing under (+)Fe and (−)Fe conditions. [Correction added on 2 December 2015, after first online publication: Some of the values in Figure 3 were incorrect and have now been amended in this current version and in Table S4 in the Supporting Information.]

In previous metabolic flux studies of *Pseudomonas* wherein metabolite labeling was deduced from amino acid labeling determined via gas chromatography‐MS, some metabolic reactions could not be determined (Fuhrer et al. 2005; del Castillo et al. 2007; Blank et al. 2008; Sudarsan et al. 2014). In particular, due to the explicit determination of the labeling patterns of free metabolites via LC‐MS analysis, we were able to discriminate the relative distribution of glucose entry routes into the central carbon metabolism. Following uptake, glucose could be entered directly into the cytosol via phosphorylation to G6P followed by phosphorylation to 6P‐Glucn or via periplasmic oxidation to gluconate and subsequent phosphorylation to 6P‐Glucn (Fig. 3A). The MFA revealed that the majority of glucose consumed (~81–91%) was first converted to gluconate prior to its conversion to 6P‐Glucn, thus contrasting the assumption that the majority of glucose is directly assimilated as G6P (Fuhrer et al. 2005; Blank et al. 2008; Sudarsan et al. 2014) (Table S4). For downstream metabolism, the normalized reaction rates (ratio of metabolic reaction rates over the glucose uptake rate) obtained with our flux analysis were generally in good agreement (within the same order of magnitude) with previous values obtained with glucose‐grown Fe‐replete *P. putida* KT2440 (Fuhrer et al. 2005; del Castillo et al. 2007; Blank et al. 2008; Sudarsan et al. 2014) (Table S4).

Our MFA of the Fe‐limited cells was based on the biomass composition previously determined for Fe‐replete *P. putida*. However, the biomass composition of Fe‐limited cells may be different due, on one hand, to the depletion in Fe‐containing components (e.g., cytochromes, Fe‐S cofactors) that are required for many enzymes and, on the other hand, to the increased expression of genes and proteins that promote Fe acquisition (Vasil and Ochsner 1999). We evaluated the sensitivity of the metabolic flux balance analysis under Fe limitation to changes in the carbon investment toward biomass (either 200% less or 50% more than the reference value). Our sensitivity analysis demonstrated that these shifts in the biomass‐invested carbons did not change the estimated metabolic reactions rates outside of their reported imprecision (Fig. S8).

With respect to the comparison between the metabolic fluxes in the Fe‐replete and Fe‐limited cells, there was an increase (by 10%) in the periplasmic oxidation of glucose to gluconate accompanied by a decrease (by up to twofold) in the metabolic fluxes throughout the intracellular metabolism under Fe limitation (Fig. 3A). This decrease in intracellular metabolic fluxes was a direct consequence of carbon losses via gluconate secretion (Fig. 3A). The reduction in the intracellular metabolic fluxes was the greatest (by more than twofold) in the nonoxidative PPP, the pyruvate shunt (malate to pyruvate and pyruvate to OAA), and from glucose to G6P (Fig. 3A). Furthermore, we found that effluxes from metabolites invested only in biomass growth were reduced by more than fourfold whereas effluxes from metabolites also invested in siderophore biosynthesis are reduced by less than twofold (Fig. 3A). These differences throughout the metabolic network were in accordance with differential investment of metabolite precursors into biomass and siderophore production.

From the MFA‐constrained estimations, we obtained the fractional amount of the carbon uptake (as glucose) that was invested toward biomass growth, siderophore secretion, CO_2_ efflux, and metabolite secretions (Fig. 3B). The model was allowed to constrain within the imprecision of one standard deviation of the averaged values of the aforementioned experimental data. In agreement with the growth rates (Fig. 1B), the investment of the carbon uptake rate toward biomass production was considerably higher in Fe‐replete cells (55.5 ± 0.2%) than in Fe‐limited cells (10.5 ± 0.01%) (Figs. 1B and 3B). The siderophore production accounted for an appreciable portion of the consumed carbon in the Fe‐limited cells (8.9 ± 1.1%), which was close to the amount of carbons invested toward biomass growth. However, as illustrated by the secretion rates (Fig. 1C), the gluconate secretion rate was the major route of carbon losses (48.8 ± 0.01%) in the Fe‐limited cells (Fig. 3B). We also estimated the possible production and secretion of 2‐ketogluconate, the oxidized product of gluconate in the periplasm, and found that this represented less than 5% of the glucose uptake under both Fe conditions (Fig. S9 and Table S5). In agreement with this estimation, the production of 2‐ketogluconate was previously proposed to be minimal when glucose or gluconate concentration is high (Tlemçani et al. 2008).

Regarding the fractional amount of the glucose uptake lost as CO_2_ efflux, this value was only slightly higher in Fe‐replete cells (37.8 ± 0.7% of glucose) than in Fe‐limited cells (31.0 ± 2.0%) (Fig. 3B). The decrease in CO_2_ efflux (only 18% reduction) under Fe limitation was not proportional to the significant difference in the amount of carbon dedicated to biomass production (about 81% less under Fe limitation). It appears that the biosynthetic demands for biomass growth in the Fe‐limited cells were replaced by those for siderophore biosynthesis such that the Fe‐limited metabolism of the assimilated glucose still resulted in a significant CO_2_ efflux.

### Monitoring *in vivo* changes in metabolic enzyme activity via kinetic ^13^C flux profiling

**Figure 4:**
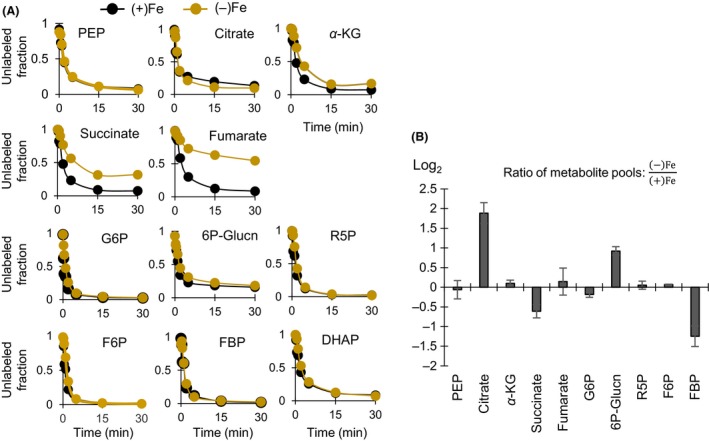
Kinetic ^13^C metabolic flux profiling and intracellular metabolite pools. (A) Kinetic incorporation of fully labeled glucose into intracellular metabolites of Fe‐limited [(−)Fe, light brown] and Fe‐replete [(+)Fe, black] cells. For clarity, only the averaged values (from four biological replicates) are shown; standard deviation values are less than 5%. (B) Changes in the intracellular pool of selected metabolites in (−)Fe cells relative to (+)Fe cells during exponential growth. Data (average ± standard deviation) were obtained from three biological replicates. Metabolite names in (A) and (B) are the same as in Figure 1.

Confirmation of the intracellular metabolic changes under Fe limitation requires monitoring of metabolic enzyme activity. Determination of enzyme activity with cell lysates uses excess substrate and assumes no rapid turnover of the product formed, the absence of metabolic regulation, and intact enzyme upon cell lysis. This approach is not therefore representative of the intracellular milieu. The KFP approach presents a practical alternative to capturing changes in cellular metabolic fluxes *in vivo* (Yuan et al. 2008). In this approach, the flux of assimilated labeled substrate through a metabolic enzyme is taken to be directly proportional to the labeling kinetics of the downstream metabolite (i.e., the product) and to the pool of the upstream metabolite (i.e., the substrate) (Yuan et al. 2008). Here, we obtained both the kinetic incorporation of [U‐^13^C_6_]‐glucose and the intracellular levels for selected intracellular metabolites (Fig. 4A and B). Quantitative flux determinations through the entire metabolic network via KFP are challenging due to the reversibility of many metabolic reactions, the requirement for labeling and pool data of all metabolites involved, and incompatibility between turnover of precursor and downstream metabolites (Yuan et al. 2008). Therefore, the KFP and MFA are often adopted as complementary approaches (Yuan et al. 2008). The KFP was used here to provide independent experimental corroboration for the changes in metabolic fluxes determined by the MFA.

Our KFP results captured decreased metabolic fluxes through several points in the TCA cycle, as well as in the ED pathway, during Fe limitation. We obtained similar labeling kinetics for both PEP (a metabolite upstream of citrate) and citrate in the *P. putida* cells under both Fe conditions (Fig. 4A). However, while the pool of PEP remained unchanged, there was a near fourfold accumulation in the citrate pool in the Fe‐limited cells (Fig. 4B). Accumulation of metabolites upstream of downregulated TCA cycle enzymes was reported in Fe‐deficient *S. cerevisiae* (Shakoury‐Elizeh et al. 2010). The active site of aconitase, the enzyme that initiates citrate metabolism in the TCA cycle, possesses an Fe‐sulfur cluster. Both genetic downregulation and decreased activity of aconitase have been reported in Fe‐limited *P. aeruginosa*,* P. fluorescens*, and *P. syringae* (Somerville et al. 1999; Vasil 2007; Kim et al. 2010; Lim et al. 2012). In accordance with these previous reports, the measured citrate buildup implied that further downstream catabolism of citrate in the TCA cycle was compromised in Fe‐limited cells. Accordingly, our MFA had determined a 75% reduction in the downstream conversion of citrate to *α*‐KG under Fe limitation (Fig. 3A).

Further along the TCA cycle downstream of citrate and *α*‐KG, there was a decrease in both labeling kinetics and metabolite levels in the Fe‐limited cells (Fig. 4A and B). Despite the accumulation of citrate, there was no significant change in the intracellular level of *α*‐KG. Furthermore, the succinate pool was depleted by 64% under Fe limitation (Fig. 4B). A slower labeling kinetics for succinate in the Fe‐limited cells confirmed that the biosynthetic flux of succinate was decreased downstream of *α*‐KG (Fig. 4A). We note that four moles of *α*‐KG and one mole of succinate are required for each mole of the siderophore PVD produced (Table S4). Therefore, the decrease in the succinate pool under Fe limitation may be a result of depletion of *α*‐KG due to siderophore biosynthesis, a decrease in the activity of the enzymes involved in the conversion of *α*‐KG to succinate, and an increase in the consumption of succinate. The relative contribution of these factors cannot be resolved by our experimental data. However, the MFA demonstrated that effluxes out of *α*‐KG (for biomass and siderophore) led to a decrease in the metabolic reaction rates downstream of *α*‐KG (Fig. 3A).

In a similar fashion, a slower labeling kinetics for fumarate when compared to succinate reflected a decreased activity of succinate dehydrogenase (Fig. 4A). This enzyme, which has four Fe‐sulfur clusters, catalyzes the conversion of succinate to fumarate in the TCA cycle (Fig. 4A). The labeling half‐time of fumarate was about eight times slower in the Fe‐limited cells (Fig. 4A). As the fumarate pools were the same in both cells, the significant delay in the labeling kinetics of fumarate under Fe limitation thus indicated that the flux through fumarate was significantly decreased under Fe limitation (Fig. 4A and B). In addition to decreased de novo ribonucleotide biosynthesis during Fe‐limited growth as previously discussed, impairement of fumarate biosynthesis could result from the depletion in the succinate pool in the Fe‐limited cells (Fig. 4B). Accordingly, the MFA‐estimated metabolic reaction rate from succinate to fumarate was 20% less in the Fe‐limited cells than in the Fe‐replete cells (Fig. 3A). However, the constant pool of fumarate under both Fe conditions implied that the consumption of fumarate was also reduced under Fe limitation (Fig. 4A and B). The MFA results demonstrated that the greatest depletion in metabolic reaction rates in the Fe‐limited metabolism was associated with the catabolism of the TCA cycle metabolites downstream of fumarate to form pyruvate and PEP (Fig. 3A). These metabolic changes facilitated the retention of carbon fluxes within the TCA cycle, presumably to meet the biosynthetic demands for biomass and siderophore precursors (Fig. 3A).

In addition to the TCA cycle, we also observed a change in the metabolic fluxes through the ED pathway in response to Fe limitation. There was no change in the labeling kinetics of metabolites upstream (G6P, Glucn, 6P‐Glucn) and downstream (R5P, FBP, pyruvate, ad DHAP) of the ED pathway (Fig. 4A). However, there was a small depletion in G6P and a threefold accumulation of 6P‐Glucn in the Fe‐limited cells (Fig. 4B). The decrease in the G6P level implied that the utilization of G6P was in excess of its formation rate under Fe limitation. In agreement with this, the MFA had presented a twofold decrease in the formation of G6P from glucose, accompanied by an increase in the oxidation of glucose to gluconate in the periplasm (Fig. 3A). The buildup in 6P‐Glucn, the phosphorylated product of gluconate in the cytosol, was indicative of a higher rate of 6P‐Glucn production than its consumption (Fig. 4B). Depletion in the FBP level further confirmed a reduction in ED pathway‐generated metabolites compared to the metabolic need of FBP under Fe limitation (Fig. 4B). These results are consistent with the decrease in nonoxidative PPP reported by the MFA as FBP is a metabolite precursor to F6P, an essential metabolite for the nonoxidative PPP (Figs. 2B and 3A). The MFA reported a near fourfold reduction in the metabolic flux through the nonoxidative PPP in the Fe‐limited cells (Fig. 3A).

In sum, changes in the KFP and the intracellular metabolite pool provided in vivo experimental evidence for a decrease in the fluxes responsible for the initial catabolism of glucose and gluconate upstream and downstream the ED pathway, in addition to the expected decrease in fluxes through Fe‐containing metabolic enzymes. These changes were confirmed by the MFA modeling results. These findings suggest that, in addition to gluconate secretion, there was a tight metabolic regulation of gluconate catabolism through the ED pathway under Fe limitation.

### Bacterial secretions and dissolution of Fe minerals

**Figure 5:**
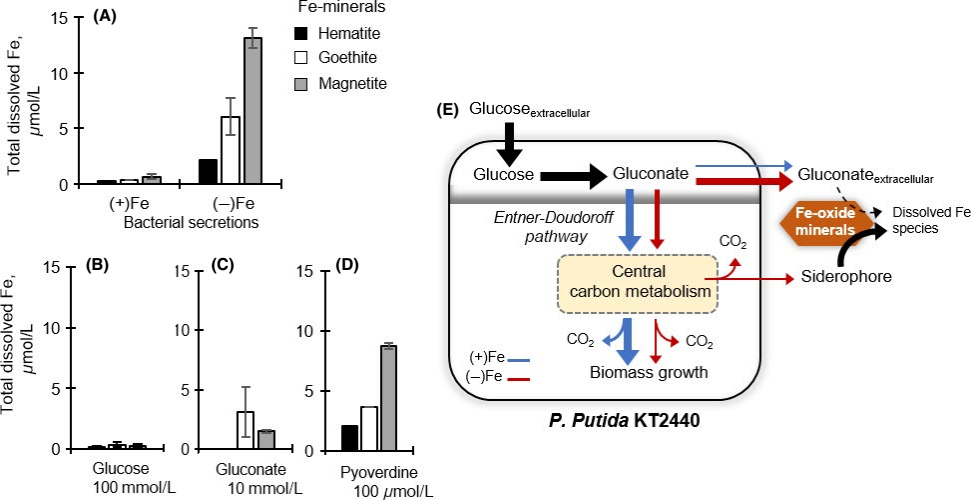
Dissolution of iron (Fe)‐bearing minerals and schematic of rerouted glucose metabolism under Fe limitation. (A) Total dissolved Fe (*μ*mol L^−1^) following reactions of (1 g L^−1^) hematite (black), goethite (white), magnetite (gray) with bacterial secretions obtained from glucose‐grown Fe‐replete [(+)Fe] and Fe‐limited [(−)Fe] *Pseudomonas putida* (A), and prepared solutions containing either 100 mmol/L glucose (Gluc) (B), 10 mmol/L gluconate (Glucn) (C), or 100 *μ*mol/L pyoverdine (PVD) (D). The error bars represent one standard error (*n* = 2–6). (E) Schematic illustration of rerouted glucose metabolism in *P. putida* KT2440 in response to Fe limitation. The catabolic route in Fe‐replete and Fe‐limited cells is shown by the blue and red lines, respectively.

Mineral dissolution by bacterial secretions has been implicated as a biogeochemical process to promote Fe availability (Reichard et al. 2007). To probe the consequence of the metabolic changes in *P. putida* in promoting Fe availability in the soil environment, we reacted the bacterial secretions (following removal of cells) with three Fe‐bearing minerals common in soils: hematite, goethite, and magnetite (Fig. 5A). We measured the total dissolved Fe concentration, which accounts for both inorganic and organic complexes of Fe in the dissolved pool of Fe. The secretions produced by the Fe‐limited cells released up to 15 *μ*mol/L Fe from the minerals whereas the secretions from the Fe‐replete cells only led to 0.64 *μ*mol/L Fe (Fig. 5A). Thus, the extracellular secretions in the Fe‐limited cells promoted, by two orders of magnitude, Fe availability from the Fe‐bearing minerals.

Due to the decrease in glucose uptake under Fe limitation, we ascertained that remaining glucose in the medium did not contribute to appreciable mineral dissolution (Fig. 5B). Reactions of the minerals with a prepared solution with 100 mmol/L glucose only resulted in 0.35 *μ*mol/L Fe in solution (Fig. 4B). Control experiments were also conducted with gluconate (10 mmol/L) and the siderophore PVD (100 *μ*mol/L). The gluconate solution facilitated dissolution of goethite and magnetite but not hematite (Fig. 5C). Gluconate‐mediated dissolution resulted in a maximum of 3 *μ*mol/L Fe, on average (Fig. 5C). At three orders of magnitude lower than the gluconate concentration, PVD was more efficient at dissolving the minerals, releasing up to 9 *μ*mol/L Fe (Fig. 5D). The increasing order of PVD‐mediated dissolution of the different minerals was similar to the dissolution obtained with Fe‐limited bacterial secretions (i.e., magnetite > goethite > hematite) (Fig. 5A and D). Thus, compared to the siderophore, the high extracellular gluconate would not contribute significantly to promoting the dissolution of the minerals (Fig. 5E). On the other hand, the carbon investment toward siderophore biosynthesis would facilitate a beneficial strategy towards enhancing Fe acquisition from minerals by soil *Pseudomonas* (Fig. 5E).

### Relevant implications and considerations

Through a combined experimental and modeling metabolomics approach, we uncovered several bypasses in glucose metabolism in Fe‐limited *P. putida* (Fig. 5E). Under Fe limitation, there was a significant reduction in the carbon influx of gluconate into the ED pathway, the primary catabolic pathway for glucose entry into the central carbon metabolism in *Pseudomonas* (Fig. 5E). This decrease in gluconate influx into intracellular metabolism was accomplished by a large increase in gluconate secretion and a decrease in the catabolism of 6P‐Glucn upstream of the ED pathway. One possible advantage of glucose oxidation to gluconate with subsequent decrease in its intracellular metabolism is the generation of reducing equivalents while decreasing the expense of processing gluconate in the Fe‐limited metabolism. [Correction added on 30 September 2015 after first online publication: This sentence has been newly added in this version]. And, consistent with reported downregulation of Fe‐containing metabolic enzymes in the TCA cycle in Fe‐limited *Pseudomonas*, we found a decrease in metabolic fluxes in the TCA cycle downstream of these enzymes. The secretion of PVD, the high‐affinity siderophore produced by *Pseudomonas* under Fe limitation, accounted for a relatively small fraction of the carbon uptake but a large fraction of carbon assimilated intracellularly due to metabolite investment in siderophore biosynthesis. The siderophore PVD was efficient at dissolving Fe from three Fe‐bearing minerals common in soils. We also found that the extracellular gluconate, though to a lesser extent, may promote dissolution of Fe minerals alongside the secreted PVD. Taken collectively, our findings revealed that bypasses in intracellular glucose metabolism were achieved by limiting carbon influx through enhanced gluconate secretion, decreasing flux through the ED pathway toward the rest of central carbon metabolism, and rerouting of metabolite precursors to siderophore production (Fig. 5E).

Several factors should be considered in terms of the relevance of these results. First, our findings report the metabolic response of carbon metabolism when Fe supply is severely limiting with respect to carbon supply. The mmol/L glucose concentration used in our experiments is compatible with microbial and plant secretions (e.g., nectar, root exudates) (Lievens et al. 2015) but exceeds *μ*mol/L carbohydrate concentrations in bulk soil solutions (Fischer et al. 2007). Second, our study focused on the transient metabolic response to limiting Fe availability and the metabolic analysis was therefore conducted on exponentially growing batch cultures. Further insights into the long‐term metabolic response at a range of Fe availability will necessitate the use of chemostat cultures. Third, secreted metabolites can be attributed to active transport of metabolites, cell leakage, and cell lysis. While cell lysis could be ruled out as the primary cause for metabolite secretions during exponential growth (Kabir et al. 2005), further examinations of the cell physiology as a function of Fe conditions are needed to distinguish the role of cell leakage versus membrane transporters in facilitating metabolite secretion. Fourth, we were able to demonstrate both that the bacterial secretions in Fe‐limited cells can promote dissolution of Fe‐bearing minerals and that Fe‐limited growth can be restored upon favorable Fe availability. It remains to be determined whether gluconate, in addition to serving as an overflow metabolic by‐product during Fe limitation, may also represent a recyclable organic substrate to be catabolized preferentially by *Pseudomonas* over ED pathway‐lacking environmental bacteria upon favorable Fe conditions. Such insight will shed light on whether gluconate secretion provides a competitive advantage to *Pseudomonas*. Of related importance, the findings presented would benefit from further studies with mutant strains to determine possible redundancies of the metabolic regulatory points identified in Fe‐limited cells. Finally, in order to gain a comprehensive understanding of the effects of Fe limitation on carbon metabolism in soil *Pseudomonas*, metabolic investigations with various types of organic substrates, including carbohydrates and noncarbohydrates, are warranted. As a significant contribution to this understanding, the present study provides a detailed account of the Fe‐limited metabolism of glucose, the most common carbohydrate in the environment (Fig. 5E).

## Conflict of Interest

None declared.

## Supporting information


**Figure S1.** Growth curves of *Pseudomonas putida* on glucose under Fe‐replete [(+)Fe] and Fe‐limited [(−)Fe] conditions. Data are from four biological replicates of batch cultures. The red square indicates the sampling time during early to mid‐exponential growth phase when data were taken to determine excretion rates and growth rate. Red arrows indicate sampling points for steady state, kinetic and intracellular quantitation experiments.
**Figure S2.** Effects of iron (Fe) addition (30 *μ*mol/L) on the growth of Fe‐limited cells; the time when Fe was added is shown. The data obtained with Fe‐limited cells before and after Fe addition are shown with the white‐filled and black‐filled squares, respectively. Data points are from independent biological replicates (*n* = 2–5).
**Figure S3.** Estimation of acetyl‐CoA (Ac‐CoA) labeling. (A) Biosynthesis of Ac‐CoA from pyruvate and (B) Biosynthesis of citrate from the combination of aspartate and Ac‐CoA. The Ac‐CoA labeling deduced from pyruvate was confirmed by the difference in the labeling patterns of aspartate and citrate.
**Figure S4.** Estimation of CO_2_ labeling biosynthesis of *N*‐carbamoyl‐aspartate from aspartate. *N*‐carbamoyl‐aspartate is formed from aspartate following the incorporation of dissolved CO_2_ and ammonia from the extracellular medium. Addition of ^13^C‐labeled carbons from aspartate to *N*‐carbamoyl‐aspartate is taken as the addition of labeled dissolved CO_2_.
**Figure S5.** Carbon mapping of intracellular metabolite labeling in the traditional tricarboxylic acid cycle and the glyoxylate shunt. Filled circles (black, gray, or red) indicate ^13^C‐labeled carbons. Red‐filled and red‐lined circles represent metabolites generated from the glyoxylate shunt; gray‐filled circles in OAA represent incorporation of labeled carbon dioxide (CO_2_) in solution. Legend for metabolite names are the same as reported in Figure 1 in the main text.
**Figure S6.** Model‐estimated (white bars) and experimentally determined (black bars) of isotopomer distributions in the carbon labeling patterns of selected metabolites in Fe‐replete *Pseudomonas putida* cells. Data presented are the average of two independent optimizations of experimental data with two model predictions, both shown with standard deviation error bars. Legend for metabolite names: G6P, glucose‐6‐phosphate; F6P, fructose‐6‐phosphate; FBP, fructose‐1,6‐bisphosphate; 3PG, 3‐phosphoglycerate; Xu5P, xylulose‐5‐phosphate; S7P, sedoheptulose‐7‐phosphate.
**Figure S7.** Model‐estimated (white bars) and experimentally determined (black bars) of isotopomer distributions in the carbon labeling patterns of selected metabolites in Fe‐limited *Pseudomonas putida* cells. Data presented are the average of two independent optimizations of experimental data with two model predictions, both shown with standard deviation error bars. Legend for metabolite names: G6P, glucose‐6‐phosphate; F6P, fructose‐6‐phosphate; FBP, fructose‐1,6‐bisphosphate; 3PG, 3‐phosphoglycerate; Xu5P, xylulose‐5‐phosphate; S7P, sedoheptulose‐7‐phosphate.
**Figure S8.** Changes in metabolic reactions rates in response to 100% decrease (lower, blue) and 50% increase (upper, green) in metabolite contribution toward biomass compared to the rates obtained from the reference biomass composition (as detailed Table S4). Model‐estimated values (averages ± standard deviations) are shown.
**Figure S9.** Estimation of 2‐ketogluconate production. The production of 2‐ketogluconate was estimated by taking into account the measured flux (r_4_) from our experiments and the estimated flux (r_4_ + r_6_) from our metabolic flux analysis for gluconate excretion. The estimated flux was only greater than the measured flux in the Fe‐replete cells (see Table S4 below). The difference is taken as a flux out of gluconate to form 2‐ketogluconate.
**Figure S10.** Effects of Fe on pyoverdine (PVD) quantification via UV–vis absorbance (400 nm). Measurements of PVD concentrations in bacterial supernatants in the absence (control) and in the presence of 50 nm or 50 *μ*mol/L of either Fe^2+^ (FeSO_4_) or Fe^3+^ (FeCl_3_). Within a precision of two standard deviation values, the quantitation of PVD was not affected by the addition of Fe higher than the concentration in the starting solution in the Fe‐replete growth medium (Fe^2+^ = 20 *μ*mol/L).
**Table S1.** Specific rates (*μ*mol g_CDW_^−1^ h^−1^) of siderophore and metabolite secretions in exponentially growing *Pseudomonas putida* KT2440 under Fe‐replete [(+)Fe] and Fe‐limiting [(‐)Fe] conditions.
**Table S2.** Carbon requirement for pyoverdine biosynthesis.
**Table S3.** Intracellular metabolic rates determined from quantitative flux modeling of the metabolism in Fe‐replete [(+)Fe] and Fe‐limited [(−)Fe] *Pseudomonas putida* using the 13CFLUX2 software. These metabolic fluxes are illustrated Figure 3A in the main text. Refer to Figure 1A in the main text for the legend of metabolite names.
**Table S4.** Normalized reaction rates (%)^1^ from metabolic flux balance analysis in Fe‐replete [(+)Fe] *Pseudomonas putida* KT2440.
**Table S5.** Estimation of gluconate flux toward 2‐ketogluconate.
**Table S6.** Metabolite effluxes toward biomass growth determined from the biomass composition of *Pseudomonas putida*.
