# Supplementary material for: Bypasses in intracellular glucose metabolism in iron‐limited Pseudomonas putida
Source: Microbiologyopen. 2015 Sep 16;5(1):3–20. doi: 10.1002/mbo3.287 (PMC4767421; doi:10.1002/mbo3.287)
Supplement: Supplementary file 1 — Figure S1. Growth curves of Pseudomonas putida on glucose under Fe‐replete [(+)Fe] and Fe‐limited [(−)Fe] conditions. Data are from four biological replicates of batch cultures. The red square indicates the sampling time during early to mid‐exponential growth phase when data were taken to determine excretion rates and growth rate. Red arrows indicate sampling points for steady state, kinetic and intracellular quantitation experiments. Figure S2. Effects of iron (Fe) addition (30 μmol/L) on the growth of Fe‐limited cells; the time when Fe was added is shown. The data obtained with Fe‐limited cells before and after Fe addition are shown with the white‐filled and black‐filled squares, respectively. Data points are from independent biological replicates (n = 2–5). Figure S3. Estimation of acetyl‐CoA (Ac‐CoA) labeling. (A) Biosynthesis of Ac‐CoA from pyruvate and (B) Biosynthesis of citrate from the combination of aspartate and Ac‐CoA. The Ac‐CoA labeling deduced from pyruvate was confirmed by the difference in the labeling patterns of aspartate and citrate. Figure S4. Estimation of CO2 labeling biosynthesis of N‐carbamoyl‐aspartate from aspartate. N‐carbamoyl‐aspartate is formed from aspartate following the incorporation of dissolved CO2 and ammonia from the extracellular medium. Addition of 13C‐labeled carbons from aspartate to N‐carbamoyl‐aspartate is taken as the addition of labeled dissolved CO2. Figure S5. Carbon mapping of intracellular metabolite labeling in the traditional tricarboxylic acid cycle and the glyoxylate shunt. Filled circles (black, gray, or red) indicate 13C‐labeled carbons. Red‐filled and red‐lined circles represent metabolites generated from the glyoxylate shunt; gray‐filled circles in OAA represent incorporation of labeled carbon dioxide (CO2) in solution. Legend for metabolite names are the same as reported in Figure 1 in the main text. Figure S6. Model‐estimated (white bars) and experimentally determined (black bars) of isotopomer distribution [file MBO3-5-003-s001.pdf]

Bypasses in Intracellular Glucose Metabolism in Iron-Limited *Pseudomonas putida*

Samantha S. Sasnow, Hua Wei, and Ludmilla Aristilde*

Department of Biological and Environmental Engineering, College of Agricultural and Life Sciences, Cornell University, Ithaca, NY 14853, USA

*Corresponding author: Department of Biological and Enviornmental Engineering, Cornell University, Ithaca, NY 14853 USA. Phone: (607) 255-6845. Fax: (607) 607.255.4449. E-mail: ludmilla@cornell.edu

SUPPLEMENTARY INFORMATION

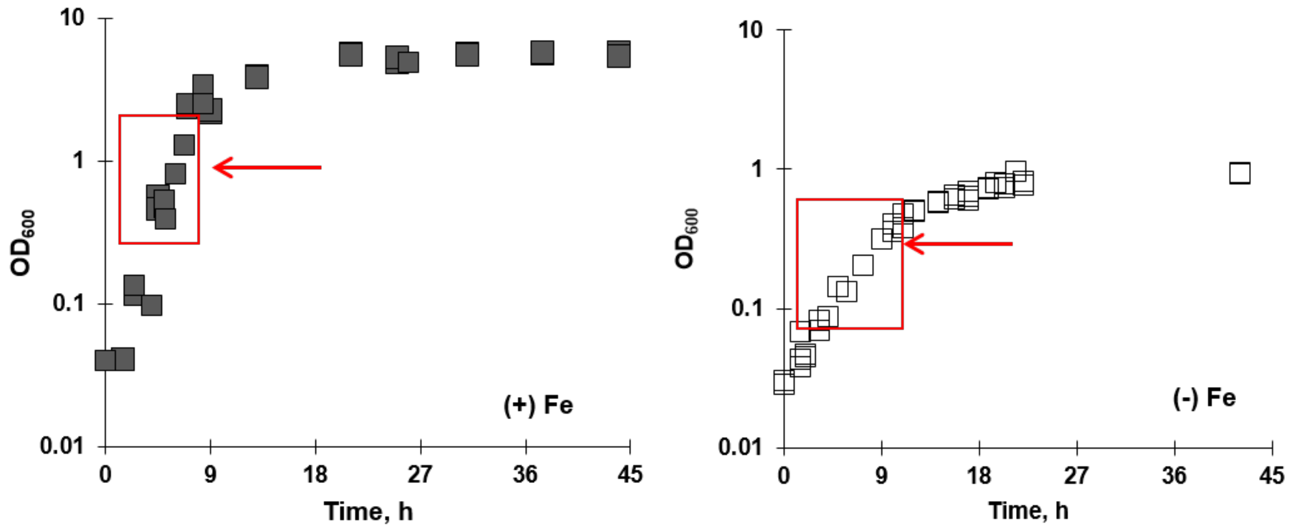

Fig. S1. Growth curves of *P. putida* on glucose under Fe-replete [(+)Fe] and Fe-limited [(-)Fe] conditions. Data are from four biological replicates of batch cultures. The red square indicates the sampling time during early to mid-exponential growth phase when data were taken to determine excretion rates and growth rate. Red arrows indicate sampling points for steady state, kinetic and intracellular quantitation experiments.

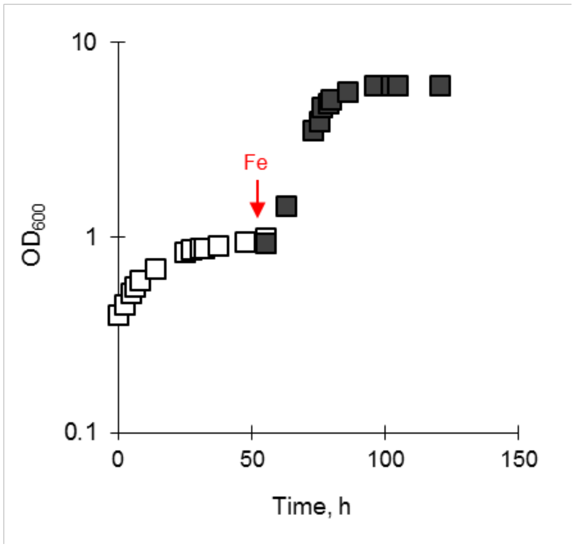

Fig. S2. Effects of iron (Fe) addition (30 μM) on the growth of Fe-limited cells; the time when Fe was added is shown. The data obtained with Fe-limited cells before and after Fe addition are shown with the white-filled and black-filled squares, respectively. Data points are from independent biological replicates (n = 2 to 5).

Table S1. Specific rates (µmol g_{CDW}^{-1} h^{-1}) of siderophore and metabolite secretions in exponentially-growing *P. putida* KT2440 under Fe-Replete [(+)Fe] and Fe-Limiting [(-)Fe] conditions.

	**(+)Fe**	**(-)Fe**	**# of carbons invested**
Siderophore Secretion			
Pyoverdine	ND	23.7 ± 4.4	62
Metabolite Secretion			
Gluconate	94.7 ± 13.9	1074.1 ± 223.0	6
3-phosphoglycerate	2.68 ± 0.52	ND	3
Pyruvate	4.67 ± 1.66	0.07 ± 0.00	3
6-phosphogluconate	--	0.115 ± 0.090	6
Phosphoenolpyruvate	0.002 ± 0.001	ND	3
Citrate	ND	4.73 ± 2.10	6
α-ketoglutarate	ND	0.018 ± 0.002	5
Glutamate	ND	0.023 ± 0.009	5
Succinate	ND	ND	4
Fumarate	0.023 ± 0.007	0.004 ± 0.000	4
Malate	0.005 ± 0.001	0.036 ± 0.017	4
Aspartate	ND	ND	4

ND: indicates that the quantified metabolites or siderophore were neither detected nor accumulated in the extracellular medium over the course of the exponential growth of the cells.

Table S2. Carbon requirement for pyoverdine biosynthesis

		# of Carbons from Metabolic Pathways		
Pyoverdine Components	Metabolite Precursors	Downstream of the Entner-Doudoroff pathway	Pentose phosphate Pathway	Tricarboxylic acid cycle
Tetradecanoic acid[a]	7 Acetyl-CoA	14		
Succinate	1 succinate			4
Chromophore (1 diaminobutyrate +1 Tyr)	1 α-ketoglutarate + 2 phosphoenolpyruvate + 1 erythrose-4-phosphate	6	4	5
Aspartate	1 oxaloacetate			4
Ornithine (1 glutamate)	1 α-ketoglutarate			5
Hydroxyl-aspartate	1 oxaloacetate			4
Diaminobutyrate (1 glutamate)	1 α-ketoglutarate			5
Glycine (1 serine)	1 3-phosphoglycerate	3		
Serine	1 3-phosphoglycerate	3		
Cyclic ornithine (1 glutamate)	1 α-ketoglutarate			5

[a]Tetradecanoic acid is attached to the pre-pyoverdine molecule that is excreted from the cytosol into the periplasm.

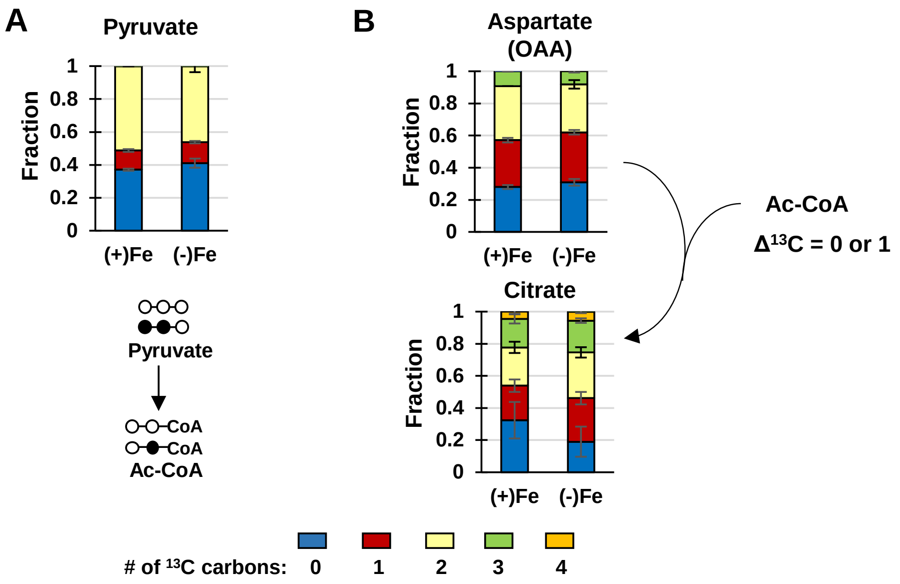

Fig. S3. Estimation of Acetyl-CoA (Ac-CoA) labeling.
Biosynthesis of Ac-CoA from pyruvate and (B) Biosynthesis of citrate from the combination of aspartate and acetyl-CoA (Ac-CoA). The Ac-CoA labeling deduced from pyruvate was confirmed by the difference in the labeling patterns of aspartate and citrate.

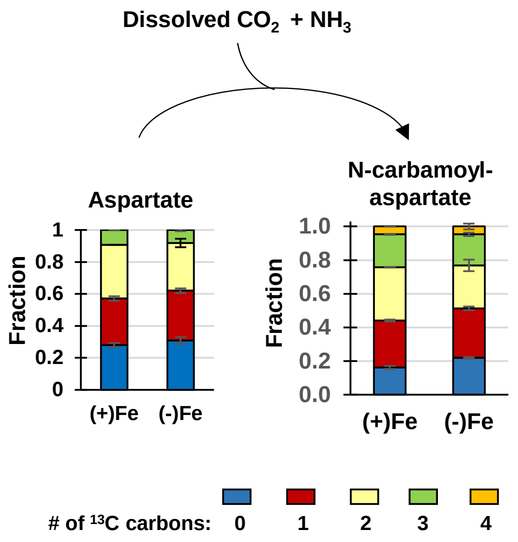

Fig. S4. Estimation of CO_2 labeling
Biosynthesis of N-carbamoyl-aspartate from aspartate. N-carbamoyl-aspartate is formed from aspartate following the incorporation of dissolved CO_2 and ammonia from the extracellular medium. Addition of ^{13}C-labeled carbons from aspartate to N-carbamoyl-aspartate is taken as the addition of labeled dissolved CO_2.

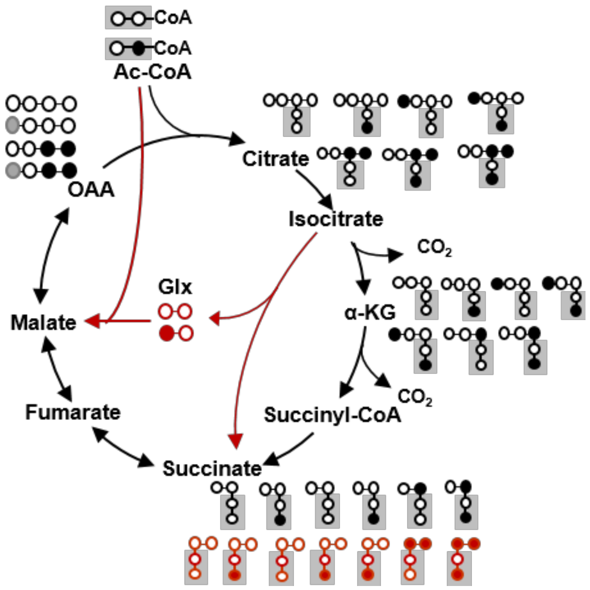

Fig. S5. Carbon mapping of intracellular metabolite labeling in the traditional TCA cycle and the glyoxylate shunt. Filled circles (black, gray, or red) indicate ^{13}C labeled carbons. Red-filled and red-lined circles represent metabolites generated from the glyoxylate shunt; gray-filled circles in OAA represent incorporation of labeled carbon dioxide (CO_2) in solution. Legend for metabolite names are the same as reported in Fig 1 in the main text.

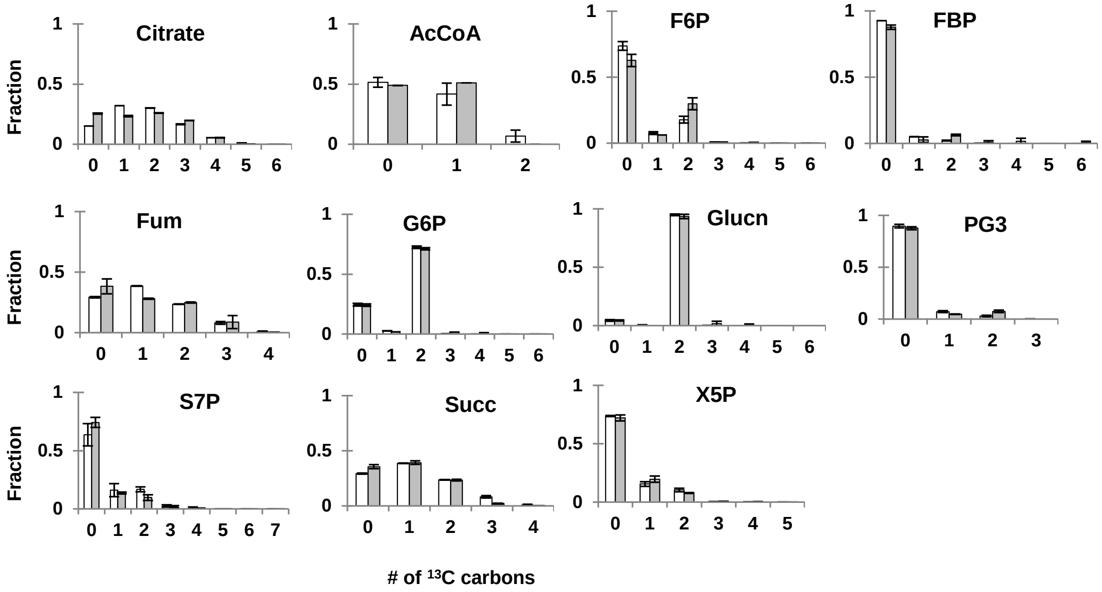

Fig. S6. Model-estimated (white bars) and experimentally-determined (black bars) of isotopomer distributions in the carbon labeling patterns of selected metabolites in Fe-replete *P. putida* cells. Data presented is the average of two independent optimizations of experimental data with two model predictions, both shown with standard deviation error bars. Legend for metabolite names: G6P, glucose-6-phosphate; F6P, fructose-6-phosphate; FBP, fructose-1,6-bisphosphate; PG3, 3-phosphoglycerate; X5P, xylulose-5-phosphate; S7P, sedoheptulose-7-phosphate; Succ, succinate; Glucn, gluconate; AcCoA, acetyl coenzyme A; Fum, fumarate.

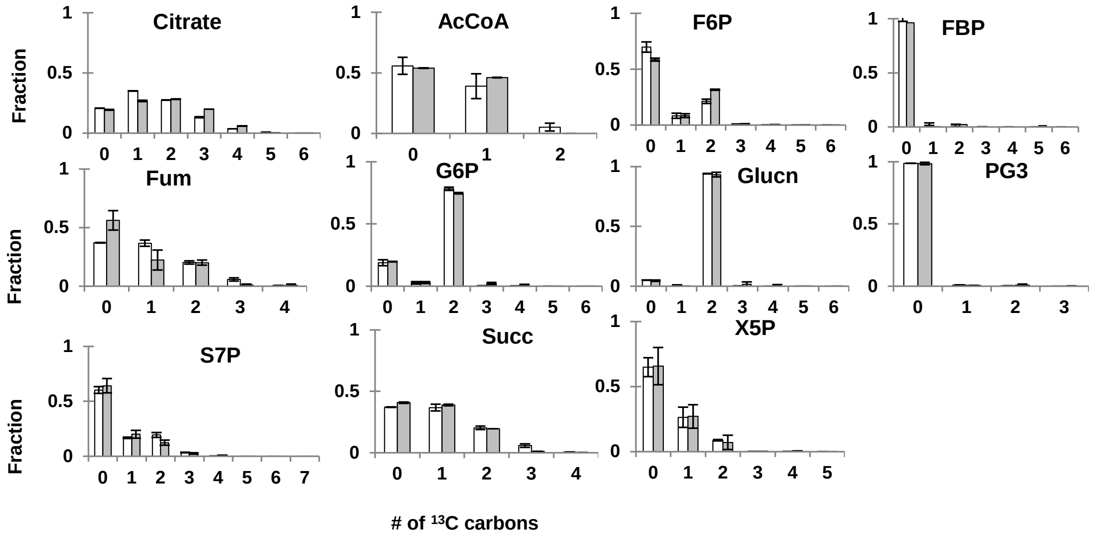

Fig. S7. Model-estimated (white bars) and experimentally-determined (black bars) of isotopomer distributions in the carbon labeling patterns of selected metabolites in Fe-limited *P. putida* cells. Data presented is the average of two independent optimizations of experimental data with two model predictions, both shown with standard deviation error bars. Legend for metabolite names: G6P, glucose-6-phosphate; F6P, fructose-6-phosphate; FBP, fructose-1,6-bisphosphate; PG3, 3-phosphoglycerate; X5P, xylulose-5-phosphate; S7P, sedoheptulose-7-phosphate; Succ, succinate; Glucn, gluconate; AcCoA, acetyl coenzyme A; Fum, fumarate.

Table S3. Intracellular metabolic rates determined from quantitative flux modeling of the metabolism in Fe-replete [(+)Fe] and Fe-limited [(-)Fe] *P. putida* using the13CFLUX2 software. These metabolic fluxes are illustrated in Fig. 6C and Fig. 6D. Refer to Fig 1 for the legend of metabolite names.

Reactions	(+)Fe	(-)Fe
	mM gCDW^{-1} h^{-1}	
$Gluc_{extracellular}$ → $Gluc_{periplasmic}$	4.71±0.30	2.46±0.20
$Gluc_{periplasmic}$ → G6P	0.91±0.12	0.23±0.20
$Gluc_{periplasmic}$→ $Glucn_{periplasmic}$	3.80±0.26	2.23±0.27
$Glucn_{periplasmic}$ → 6P-Glucn	3.67±0.25	1.03±0.22
G6P → 6P-Glucn	1.62±0.22	0.50±0.12
G6P → F6P	-0.80±0.31	-0.29±0.09
FBP → F6P	0.96±0.32	0.29±0.09
DHAP + GAP → FBP	0.96±0.32	0.29±0.09
GAP → 3-PG	3.02±0.41	0.89±0.11
3-PG → PEP	2.55±0.41	0.79±0.11
PEP → Pyr	2.48±0.34	0.77±0.11
6P-Glucn → Pyr+GAP	5.22±0.43	1.51±0.15
6P-Glucn → R5P	0.037±0.004	0.016±0.002
6P-Glucn → Xu5P	0.037±0.004	0.015±0.002
GAP+S7P → Xu5P+R5P	0.024±0.003	0.019±0.002
GAP+S7P → E4P+F6P	0.024±0.003	0.019±0.002
E4P+Xu5P → F6P+GAP	-0.18±0.01	-0.026±0.002
Pyr → Acetyl-CoA+CO_2	4.58±0.29	1.75±0.17
OAA+Acetyl-CoA → Citrate	4.13±0.27	1.63±0.17
Citrate → α-KG	4.11±0.28	1.62±0.17
α-KG → Succinate+CO_2	3.50±0.24	1.47±0.16
Succinate → Fumarate	3.51±0.24	1.45±0.16
Fumarate → Malate	3.57±0.24	1.46±0.16
Malate → OAA	1.79±0.18	1.32±0.15
Malate → Pyr	1.65±0.25	0.14±0.01
Pyr+CO_2 → OAA	3.59±0.26	0.52±0.04
OAA → PEP+CO_2	0.35±0.10	0.07±0.01
OAA → Fumarate	0.060±0.004	7.6±0.7E-3

Table S4. Normalized reaction rates (%)[1] from metabolic flux balance analysis in Fe-replete [(+)Fe] *P. putida* KT2440.

Fe conditions	(+)Fe	(+)Fe	(+)Fe	(+)Fe	(+)Fe	(-)Fe
Source	Fuhrer et al., 2005	del Castillo et al., 2007	Blank et al., 2008	Sudarsan et al., 2014	Present study	Present study
$Gluc_{periplasmic}$ → G6P	100 ± 7.5	NR	100 ± 3	100 ± 2.3	19.3 ± 2.3	9.2 ± 8.0
$Gluc_{periplasmic}$ → $Glucn_{periplasmic}$	NR	NR	NR	NR	80.6 ± 2.3	90.8 ± 8.0
$Glucn_{periplasmic}$ → 6P-Glucn	NR	NR	NR	NR	78.0 ± 1.9	42.0 ± 8.0
G6P → 6P-Glucn	104.8 ± 9.4	NR	NR	99.5 ± 5.4	34.3 ± 4.0	20.5 ± 4.4
6P-Glucn → Xu5P + CO_2	2.7 ± 0.6[2]	NR	5.5 ± 0.5[2]	1.3 ± 0.4[2]	0.8 ± 0.1	0.6 ± 0.1
6P-Glucn → R5P + CO_2	2.7 ± 0.6[2]	NR	5.5 ± 0.5[2]	1.3 ± 0.4[2]	0.8 ± 0.1	0.6 ± 0.0
Xu5P → R5P	NR	NR	NR	NR	4.1 ± 0.1	0.9 ± 0.0
GAP and S7P → Xu5P and R5P	-0.9 ± 0.3	NR	NR	0.5 ± 0.5	0.5 ± 0.0	0.7 ± 0.0
GAP and S7P → E4P and F6P	0.9 ± 0.3	NR	NR	0.5 ± 0.5	0.5 ± 0.0	0.7 ± 0.0
F6P and GAP → E4P and Xu5P	2.0 ± 0.3	NR	NR	0.0 ± 0.5	3..8 ± 0.1	1.0 ± 0.0
F6P → G6P	5.3 ± 3.1	NR	0 ± 3	0 ± 5	17.0 ± 6.5	11.6 ± 3.7
FBP → F6P	NR	NR	NR	0 ± 5	20.4 ± 6.6	11.9 ± 3.6
DHAP + GAP → FBP	6.6 ± 3.3	NR	0 ± 3	0 ± 5	20.4 ± 6.6	11.9 ± 3.6
GAP → DHAP	NR	NR	NR	NR	22.4 ± 6.7	12.2 ± 3.6
6P-Glucn → Pyruvate + GAP	99.6 ± 9.5	NR	89 ± 5	96.8 ± 5.4	110.7 ± 5.7	61.2 ± 3.6
GAP → 3-PG	83.5 ± 6.1	NR	86 ± 2	96.4 ± 5.4	64.0 ± 7.7	36.0 ± 3.6
3-PG → PEP	71.2 ± 5.7	NR	68 ± 2	88.7 ± 5.4	54.1 ± 8.0	32.1 ± 3.6
PEP → Pyruvate	81.2 ± 7.0	NR	71 ± 2	100 ± 6.3	52.7 ± 6.3	31.3 ± 3.6
Pyruvate → Acetyl-CoA + CO_2	119.5 ± 13.8	NR	90 ± 7	146.2 ± 9.9	97.3 ± 0.3	71.1 ± 3.8
Pyruvate + CO_2 → OAA	85.0 ± 8.4	78.0 ± 3.9[3]	57 ± 4	33.0 ± 6.8	76.3 ± 2.4	21.0 0.4
OAA + Acetyl-CoA → Citrate	102.7 ± 13.8	100	NR	134.8 ± 10.9	87.7 ± 1.7	66.1 ± 4.2
Citrate → α-KG + CO_2	102.7 ± 13.8	NR	68 ± 7	134.8 ± 16.7	87.3 ± 1.9	66.0± 4.1
α-KG → Succinate + CO_2	92.6 ± 13.8	NR	NR	126.7 ± 18.1	74.2 ± 1.8	59.7 ± 4.1
Succinate → Fumarate	NR	NR	55 ± 7	126.7 ± 12.2	74.5 ± 1.6	59.0 ± 4.2
Fumarate → Malate	NR	NR	NR	NR	75.8 ± 1.6	59.3 ± 4.2
Malate → OAA	45.7 ± 9.3	40.3 ± 2.0[3]	37 ± 3	126.7 ± 10.4	38.1 ± 3.0	53.4 ± 4.5
Citrate → Glyx + Succinate	NR	NR	NR	0.0 ± 8.6	0.3 ± 0.2	0.0± 0.0
Glyx + Acetyl-CoA → Malate	NR	NR	NR	NR	0.3 ± 0.2	0.0 ± 0.0
Malate → Pyruvate and CO_2	46.9 ± 7.0	40.7 ± 2.0[3]	18 ± 2	NR	35.1 ± 4.8	5.9 ± 0.3
OAA → PEP and CO_2	16.0 ± 2.3	NR	18 ± 5	15.8 ± 1.4	7.5 ± 2.0	2.9 ± 0.0
OAA → Fumarate	NR	NR	NR	NR	1.3 ± 0.0	0.31 ± 0.01

[1]Reaction rates were normalized to the glucose uptake rate. Metabolite names are the same as shown in Fig. 1.
[2]This represents half of the summed reaction rate from 6P-Glucn to PPP reported in the previous studies.
[3]The 95% confidence intervals were reported to be within 5% of the averaged values. NR: indicates that the rates of these metabolic reactions were not reported.

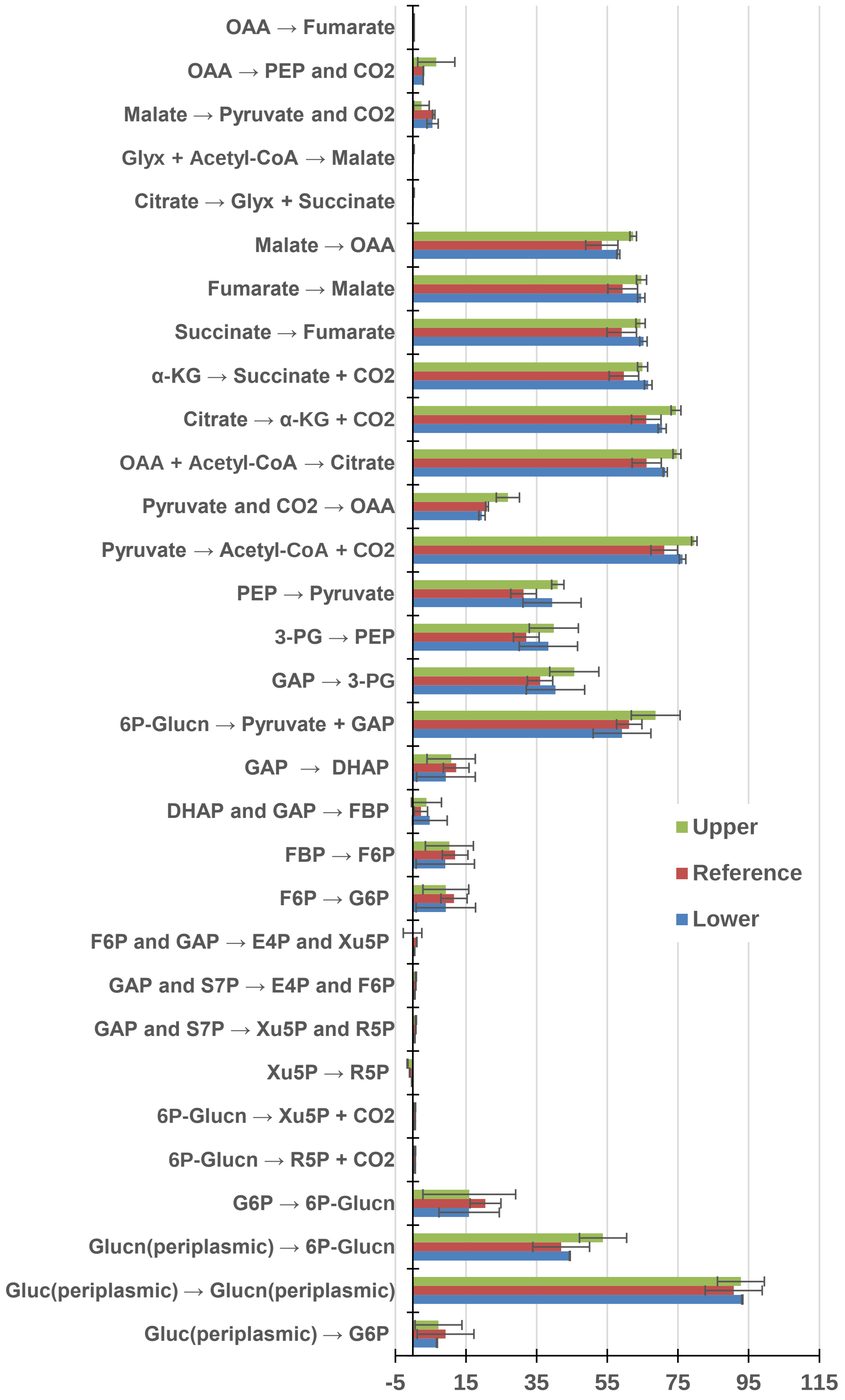

Fig. S8. Changes in metabolic reactions rates in response to 100% decrease (lower, blue) and 50% increase (upper, green) in metabolite contribution towards biomass compared to the rates obtained from the reference biomass composition (as detailed Table S4). Model-estimated values (averages ± standard deviations) are shown.

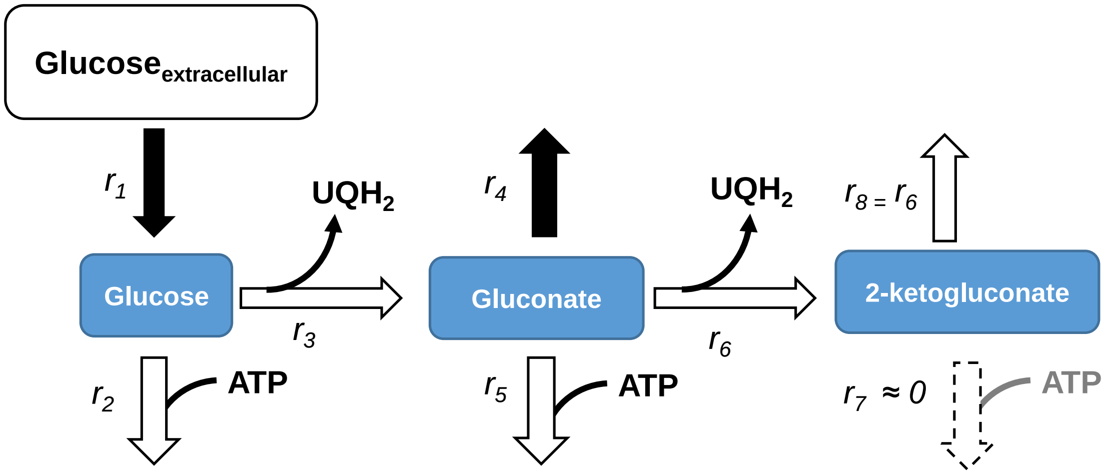

Fig S9. Estimation of 2-ketogluconate production
The production of 2-ketogluconate was estimated by taking into account the measured flux (r_4) from our experiments and the estimated flux (r_4 + r_6) from our metabolic flux analysis for gluconate excretion. The estimated flux was only greater than the measured flux in the Fe-replete cells (see Table S4 below). The difference is taken as a flux out of gluconate to form 2-ketogluconate. The estimate r_6 flux was taken into account in the estimating the total amount of reduced ubiquinone formed in the periplasm from the oxidation of glucose and gluconate.

Table S5. Estimation of gluconate flux towards 2-ketogluconate

**Fe conditions**	**(+)Fe**	**(-Fe)**
	**(μmol g_{CDW}^{-1} h^{-1})**	**(μmol g_{CDW}^{-1} h^{-1})**
Measured gluconate excretion (r_4)	94.7 ± 13.9	1074.1 ± 223.0
Model-estimated 'excretion' out of gluconate (r_4 + r_6)	125.53 ± 22.0	1201.3 ± 97.1
Difference (estimated r_6)	30.8 ± 8.1	127.2 ± 125.9

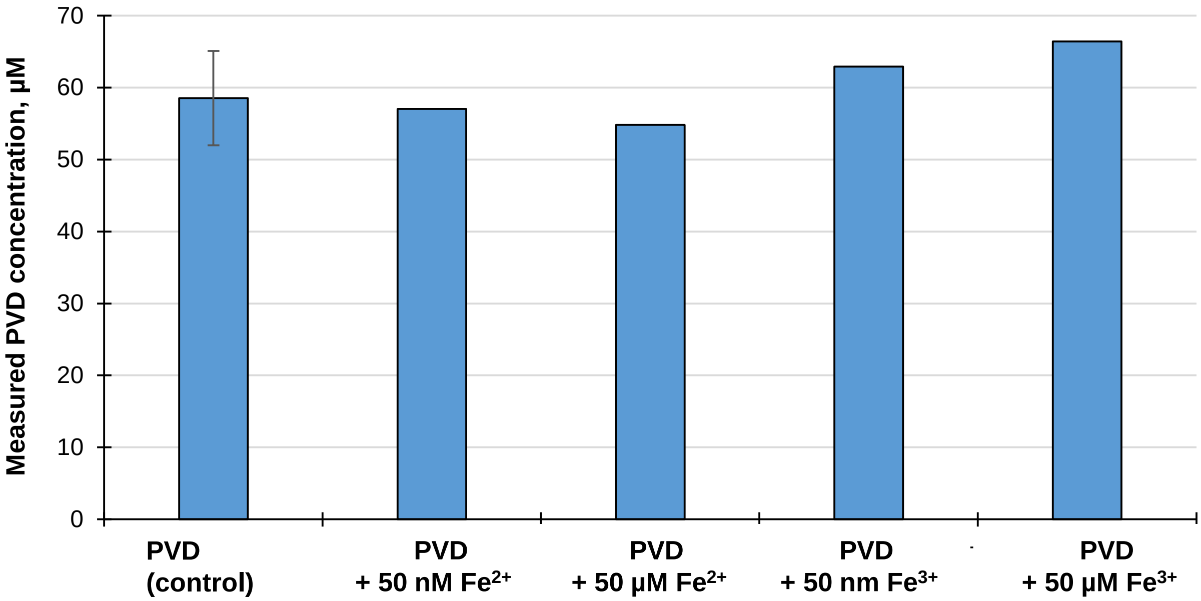

Fig. S10. Effects of Fe on pyoverdine (PVD) quantification via UV-vis absorbance (400 nm). Measurements of PVD concentrations in bacterial supernatants in the absence (control) and in the presence of 50 nm or 50 µM of either Fe^{2+} ($FeSO_4$) or Fe^{3+} ($FeCl_3$). Within a precision of two standard deviation values, the quantitation of PVD was not affected by the addition of Fe higher than the concentration in the starting solution in the Fe-replete growth medium (Fe^{2+} = 20 µM)

Table S6. Metabolite effluxes towards biomass growth determined from the biomass composition of *Pseudomonas putida*.

	Biomass Precursors										
	G6P	F6P	R5P	E4P	GAP (DHAP)	3PG	PEP	Pyr	Acetyl-CoA[1]	OAA	α-KG
	(mmol $gCDW^{-1}$)										
P. putida [2]	0.18	0	0.39	0.4	0.17	0.91	0.82	1.78	--	1.5	1.11

[1] There was no constraint on the acetyl-CoA efflux in the Fe-replete cells; the model determined this value during the flux balance analysis. In the flux balance analysis in the Fe-limited cells, the rate of acetyl-CoA invested for the pre-pyoverdine production was used as lower-bound value.

[2] Biomass composition obtained from van Duuren, 2013

Van Duuren J, Puchalka J, Mars AE, Bucker R, Eggink G, Wittmann C, Martins dos Santos V. 2013. Reconciling in vivo and in silico key biological parameters of *Pseudomonas putida* KT2440 during growth on glucose under carbon-limited condition. BMC Biotechnol. **13**(93).
